# A Simple Model Relating Gauge Factor to Filler Loading in Nanocomposite Strain Sensors

**DOI:** 10.1021/acsanm.1c00040

**Published:** 2021-03-05

**Authors:** James R. Garcia, Domhnall O’Suilleabhain, Harneet Kaur, Jonathan N. Coleman

**Affiliations:** † School of Physics, CRANN & AMBER Research Centres, Trinity College Dublin, Dublin 2, Ireland

**Keywords:** piezoresistor, strain sensor, graphene, nanotube, tunnelling

## Abstract

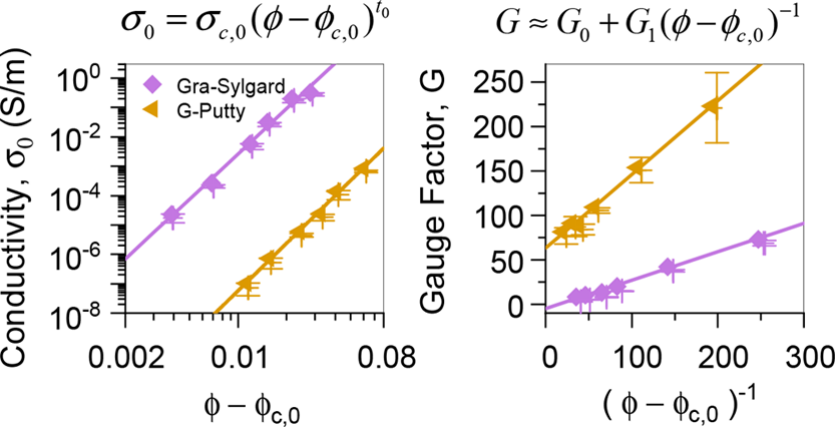

Conductive nanocomposites are often piezoresistive, displaying significant changes in resistance upon deformation, making them ideal for use as strain and pressure sensors. Such composites typically consist of ductile polymers filled with conductive nanomaterials, such as graphene nanosheets or carbon nanotubes, and can display sensitivities, or gauge factors, which are much higher than those of traditional metal strain gauges. However, their development has been hampered by the absence of physical models that could be used to fit data or to optimize sensor performance. Here we develop a simple model which results in equations for nanocomposite gauge factors as a function of both filler volume fraction and composite conductivity. These equations can be used to fit experimental data, outputting figures of merit, or predict experimental data once certain physical parameters are known. We have found these equations to match experimental data, both measured here and extracted from the literature, extremely well. Importantly, the model shows the response of composite strain sensors to be more complex than previously thought and shows factors other than the effect of strain on the interparticle resistance to be performance limiting.

## Introduction

Incipient technological developments such as the Internet of things, smart cities, and personalized medicine will require everything from packaging to buildings to clothing to gather, process, and exchange information. A critical part of this revolution will involve the proliferation of sensors and sensing technologies.^1,2^ Of the different sensor types, strain and pressure sensors are conceptually simple and have applications in a range of areas including biomedical sensing, sports performance monitoring, and robotics.^3,4^ Such sensors are usually based on piezoresistive materials which display changes in electrical resistance as they are deformed.

While an effective strain sensor must display a number of appropriate characteristics (e.g., sufficient working range,^5^ reasonable conductivity, lack of frequency or rate dependence^6^), probably the most important property is the sensitivity or gauge factor, *G*. This figure of merit is defined via Δ*R*/*R*_0_ = *Gε*, where Δ*R*/*R*_0_ is the fractional resistance change in response to an applied strain, ε. Strictly speaking, this equation applies at low strains, where Δ*R*/*R*_0_ varies linearly with strain such that *G* has a well-defined value. By far the most common type of strain sensors are metal foil strain gauges, which are cheap and effective and are widely used in industry. However, they have some significant drawbacks, namely, relatively small gauge factors of *G* ∼ 2.^7^ Such a small value means a very limited sensitivity and has driven the research community to search for sensing platforms that display much higher sensitivities.

To resolve this issue, many researchers have turned to materials science and, in particular, polymer nanocomposites. Such composites are widely used due to their processability, flexibility, and low cost. Extensive research has underlined the versatility of polymer nanocomposites where materials can be tailored to display a range of desired properties such as excellent conductivity, mechanical reinforcement, electromagnetic interference shielding, enhanced thermal stability, and polymer self-healing.^8−12^ Piezoresistive nanocomposites have proved particularly promising in the area of strain sensing as they display impressive and tunable sensitivities which far surpass current commercially available strain sensors.^5,13^ These nanocomposites contain conductive nanoparticles dispersed within an insulating matrix, typically a polymer. While nanocomposites utilizing fillers such as graphene^14−18^ and CNTs^19−21^ have been widely investigated, piezoresistive behavior has been observed with a host of other nanofillers such as conductive polymers,^22^ carbon black,^23,24^ silver nanowires,^25^ carbon fibers,^26^ and semiconducting materials.^27,28^

The electrical properties of conductive composites are understood in great detail, with much experimental and theoretical work (e.g., via percolation theory) having been carried out in recent years.^17,29^ As a result, one might imagine that the piezoresistive properties of such composites would be well understood. However, this is not the case. Although it is generally believed that the effect of strain on interparticle tunnelling is responsible for the piezoresistive effect in nanocomposites,^19,30−32^ good analytical models are not available. A number of numerical studies on the piezoresistive response in nanocomposites exist,^19,25,31^ with the recent work of Liu et al. allowing a visualization of the conductive network evolution under tensile strain.^33,34^ However, these studies are filler specific and cannot be universally applied. What are needed are straightforward models that yield simple equations, which describe how *G* varies with filler volume fraction, ϕ or indeed composite conductivity. Such models would allow the fitting of experimental data and give an insight into the nanocomposite properties that determine the gauge factor. Ultimately, this would allow for the development of nanocomposites with enhanced values of *G*.

In this study, we use percolation theory to develop a simple model relating the gauge factor (*G*) of nanocomposite piezoresistive materials to both the filler volume fraction (ϕ) and the zero-strain conductivity, σ_0_. This model’s accuracy is tested against a number of composites with different conductive fillers and is supported by a detailed study of how percolation parameters vary with applied strain.

## Results and Discussion

### Electrical Percolation

By now, much is known about the conductivity in nanocomposites. Most importantly, they show large increases in conductivity as filler loading is increased. As ϕ is increased above a critical value, the percolation threshold (ϕ_c_), the first continuous conductive paths spanning the length of the sample are formed, allowing current to flow. Above the percolation threshold (i.e., ϕ > ϕ_c_), the conductivity is usually described using classical percolation theory,^35−37^ which in its simplest form yields
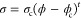
1where σ_c_ is a constant and *t* is the percolation exponent.

Many papers have been devoted to understanding the nature of ϕ_c_, σ_c_, and *t*. In a random network of nonspherical particles such as nanotubes or graphene sheets, the percolation threshold is expected to be roughly equal to the ratio of small to large particle dimensions (e.g., nanosheet thickness over length).^38,39^ However, if the particle orientation distribution becomes less random, for example, if the particles become aligned, ϕ_c_ can increase significantly.^40−42^ In addition, σ_c_ has been linked to the junction resistance between particles,^38^ which may be controlled by tunnelling^15^ or hopping,^43^ and so is sensitive to interparticle separation.

The percolation exponent, *t*, was originally expected to take universal values of *t*_un_ = 1.3 or 2 depending on whether the system in question was two- or three-dimensional.^44^ However, multiple experimental observations of *t* > 2 have led to theoretical work that shows that *t* can indeed be larger than the universal value, *t*_un_. The current consensus is that, where there is a distribution of interparticle resistances, *t – t*_un_ is controlled by the width of the interparticle junction resistance distribution.^44−46^ In polymer-based composites, we would expect disordered regions of polymer to separate the conductive particles leading to a distribution of interparticle separations and so resistances, resulting in values of *t* > 2.^29^

### Model Development

**Figure 1:**
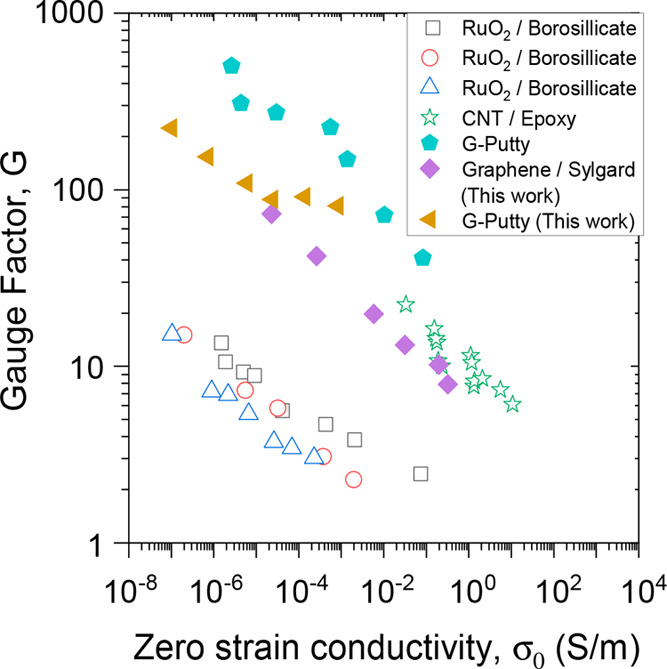
Literature data^15,19,48^ as well as data collected here for gauge factor (*G*) as a function of zero-strain conductivity (σ_0_) for a range of nanocomposites (see the Supporting Information for more data). All plots indicate an approximate power law relationship between these parameters.

Any model that describes the performance of nanocomposite strain sensors must be consistent with experimental data which means it must display certain general features. For example, almost all reports in the literature suggest that the gauge factor is highly dependent on the loading of conductive filler, with the gauge factor decreasing rapidly as the filler volume fraction is increased above the percolation threshold.^15,19^ This means that an increasing gauge factor is associated with decreasing conductivity as reported in a number of papers.^15,19,47−53^ A review of the literature identified various papers that reported both conductivity (at zero-strain) and gauge factor for various composites each measured at a range of filler loadings.^15,19,50^ As shown in Figure 1, these data sets suggest a roughly power law relationship between gauge factor (*G*) and zero-strain conductivity (σ_0_). Any successful model describing nanocomposite strain sensors must describe the dependence of *G* on both ϕ and σ_0_ in a way that is consistent with these findings and be applicable across the broad range of strain-sensing materials.

When a material is strained, the resistance changes for two reasons. First, there is a relatively small change due to the effect of strain on the sample dimensions—this is dominant in metallic strain gauges. Second, the resistance can change due to variations in the material conductivity with strain.^7^ The second effect can be positive^15,16^ or negative^28^ and can be very large in some systems,^15^ including nanocomposites.

These effects can be quantified in a simple model which shows that (see refs (3 and 15) and the Supporting Information)
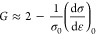
2where the subscript zero means the quantity must be taken in the limit of low strain such that, for example, σ_0_ denotes the zero-strain conductivity. This low-strain condition comes from approximations in the derivation that are valid only at low strain (see the Supporting Information).

This equation can be applied to percolative systems by differentiating eq 1 with respect to strain, assuming ϕ_c,_ σ_c_, and *t* all depend on strain. Performing this differentiation (see the Supporting Information) yields
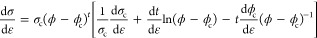
3This equation can be combined with eq 2 by taking all parameters in their low-strain limits. In that limit, σ≃σ_0_ and by extension σ_c_≃σ_c,0_, ϕ_c_≃ ϕ_c,0_, and *t≃t*_0_. This gives
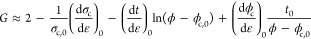
4aThis equation links the nanocomposite gauge factor to both the zero-strain percolation parameters σ_c,0_, ϕ_c,0_, and *t*_0_ as well as their derivatives, measured at low strain, (dσ_c_/dε)_0_, (dϕ_c_/dε)_0_, and (d*t*/dε)_0_. An important feature of this equation is that *G* scales with (ϕ – ϕ_c,0_)^−1^ which would explain the experimental observation that *G* increases sharply as the percolation threshold is approached from above.

We note that, because of the way eq 1 is generally used to fit conductivity versus φ data (plotting ln σ_0_ versus ln(ϕ – ϕ_c,0_) to obtain ln σ_c,0_ and *t*_0_ as fit parameters), some researchers will prefer to write eq 4a as
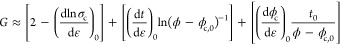
4bWe can use eq 4b to generate an equation for *G* in terms of the zero-strain conductivity, σ_0_, by noting that at zero-strain, eq 1 shows that σ_0_ = σ_c,0_(ϕ – ϕ_c,0_)^*t*_0_^, leading to
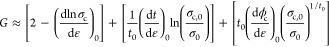
4cWe have written these equations in what might seem an unusual fashion for two reasons: to clearly differentiate the three square-bracketed terms and to facilitate a clear discussion of the sign of each term (see below). As these equations make clear, there are a number of factors, embodied by the three square-bracketed terms, which contribute to the gauge factor. We will consider these factors below.

### The Factors Contributing to the Gauge Factor

One advantage of this approach is that our understanding of electrical percolation can be used to understand nanocomposite piezoresistivity. The factors influencing the percolation parameters, ϕ_c,0_, σ_c,0_, and *t*_0_, have been outlined above. Clearly, such factors will influence *G* via eqs 4b and 4c. For example, the dispersion state of the filler will affect ϕ_c_,_0_, with filler alignment^42^ leading to higher ϕ_c,0_ compared to randomly oriented^54^ systems. According to eq 4b, reducing ϕ_c,0_ should lead to a smaller gauge factor for a given ϕ. Polymer morphology will also have an impact. If the polymer tends to crystallize on the nanofiller surface, this will increase the interparticle resistance, thus^38^ decreasing σ_c,0_. Equation 4c shows that this will reduce *G* at a given compsoite conductivity. It is worth considering how the other parameters in eqs 4b–4c impact *G*.

The first term in eqs 4b–4c is dominated by the rate of change of ln σ_c_ with ε (at low strain), (dln σ_c_/dε)_0_. To understand this term, we note that in most (but not all)^28^ polymer-based nanocomposites, where the fillers are highly conductive, we expect σ_c_ to be limited by the junction resistance (*R*_J_) between filler particles such that σ_c_ ∝ 1/*R*_J_.^38^ Whether the intersheet transport is via hopping or tunnelling,^15,43^ we expect *R*_J_ ∝e^*kd*^, where *d* is the interparticle separation (at a given strain) and *k* is a (positive) constant. Assuming that, on application of tensile strain, the interparticle separation scales with sample length, and then ε = (*d* – *d*_0_)/*d*_0_, where *d*_0_ is the zero-strain interparticle separation. Combining these ideas gives dln σ_c_/dε = −*kd*_0_, showing that (dln σ_c_/dε)_0_ should be negative (unless the interparticle charge transfer is not rate limiting^28^). This negative sign is expected and is in line with many studies showing composites to become more resistive upon tensile deformation.^15,16,19,25,31,55^ This negative sign is significant as it means the first square-bracketed term in eqs 4b and 4c must be positive.

Thus, the first term in eqs 4b–4c is predominantly associated with interparticle transport and describes the mechanism traditionally used to describe the piezoresistive response in nanocomposites.^19,30−32^ However, the existence of the two other terms in eqs 4b–4c shows that this is not the only contribution to *G*.

The second term in eqs 4b–4c is controlled by the strain dependence of the percolation exponent, (d*t*/dε)_0_. A number of papers have shown that the value of *t* is controlled by the width of the distribution of interparticle junction resistances.^44−46^ Then, the strain would most likely increase each individual junction separation according to *d* = *d*_0_(ε + 1), thus broadening the distribution and increasing *t*. Alternatively, strain should align the particles somewhat, making the network more 2D and less 3D and so reducing *t*.^32^ Thus, we expect the second term to be controlled by a combination of junction and network effects, each driving (d*t*/dε)_0_ in different directions. We note that theoretical calculations have suggested that (dln σ_c_/dε)_0_ ∝ (d*t*/dε)_0_ such that both parameters have the same sign.^46^ If this were true it would mean that (d*t*/dε)_0_ is generally negative. This is important as it means that the second term in eqs 4b–4c should be negative. This has important implications as we will discuss below.

The third term in eqs 4b–4c is controlled by the strain dependence of the percolation threshold, (dϕ_c_/dε)_0_. It is likely that the application of strain alters the structure of the network, for example by inducing particle alignment, in a way that modifies the percolation threshold. To see this, consider a composite exactly at the percolation threshold. Then, all current flows through a single conducting path with a well-defined bottleneck (where all current flows through a single interparticle junction). While the application of tensile strain would be very unlikely to create new paths, it is likely that the strain-induced change to the network structure will break the bottleneck. This will destroy the single conducting path and shift the percolation threshold (at that strain) to a higher filler volume fraction. Thus, we always expect (dϕ_c_/dε)_0_ to be positive. This is important as it means the third term in eqs 4b and 4c must always be positive. This is consistent with previous studies that investigated the effect of carbon nanotube (CNT) alignment on film conductivity (with alignment acting as a proxy for tensile strain). Such studies show that as alignment increases the percolation threshold similarly increases.^40,41^

Thus, in addition to the effect of strain on interparticle transport, which is usually considered as the mechanism controlling *G*, there are other factors related to both properties of the junctions and the network which contribute to *G*. Later in this article, we will consider the relative magnitude of these effects. However, first it is necessary to demonstrate that the equations above can actually describe experimental data.

### Using This Model to Fit Experimental Data

While eqs 4b and 4c provide a theoretical description of the dependence of *G* on ϕ and σ_0_, respectively, neither equation can be used in its current form for fitting experimental data, simply because they both contain too many fit parameters. However, they can both be simplified by considering the contributions to the second and third terms of eqs 4b–4c. Since ln(ϕ – ϕ_c,0_) is a slowly varying function compared to (ϕ – ϕ_c,0_)^−1^ as ϕ → ϕ_c,0_, it is likely that the second term of eqs 4b–4c is relatively slowly varying compared to the third. This allows us to consider the second term constant for fitting purposes, assuming that (d*t*/dε)_0_ is not much larger than *t*_0_(dϕ_c_/dε)_0_ (this will be justified further below). Applying this approximation to both eqs 4b and 4c and grouping terms allow us to write equations for *G* in the form
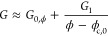
5a
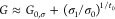
5bwhere ϕ_c,0_, *G*_0,ϕ_, *G*_1_, *G*_0,σ_, and σ_1_ are (near) constants. To maximize *G* requires all of these constants except ϕ_c,0_ to be large and positive. We note that this model implies that *G* has a near power law relationship with σ_0_, which is consistent with the observations in Figure 1.

**Scheme 1:**
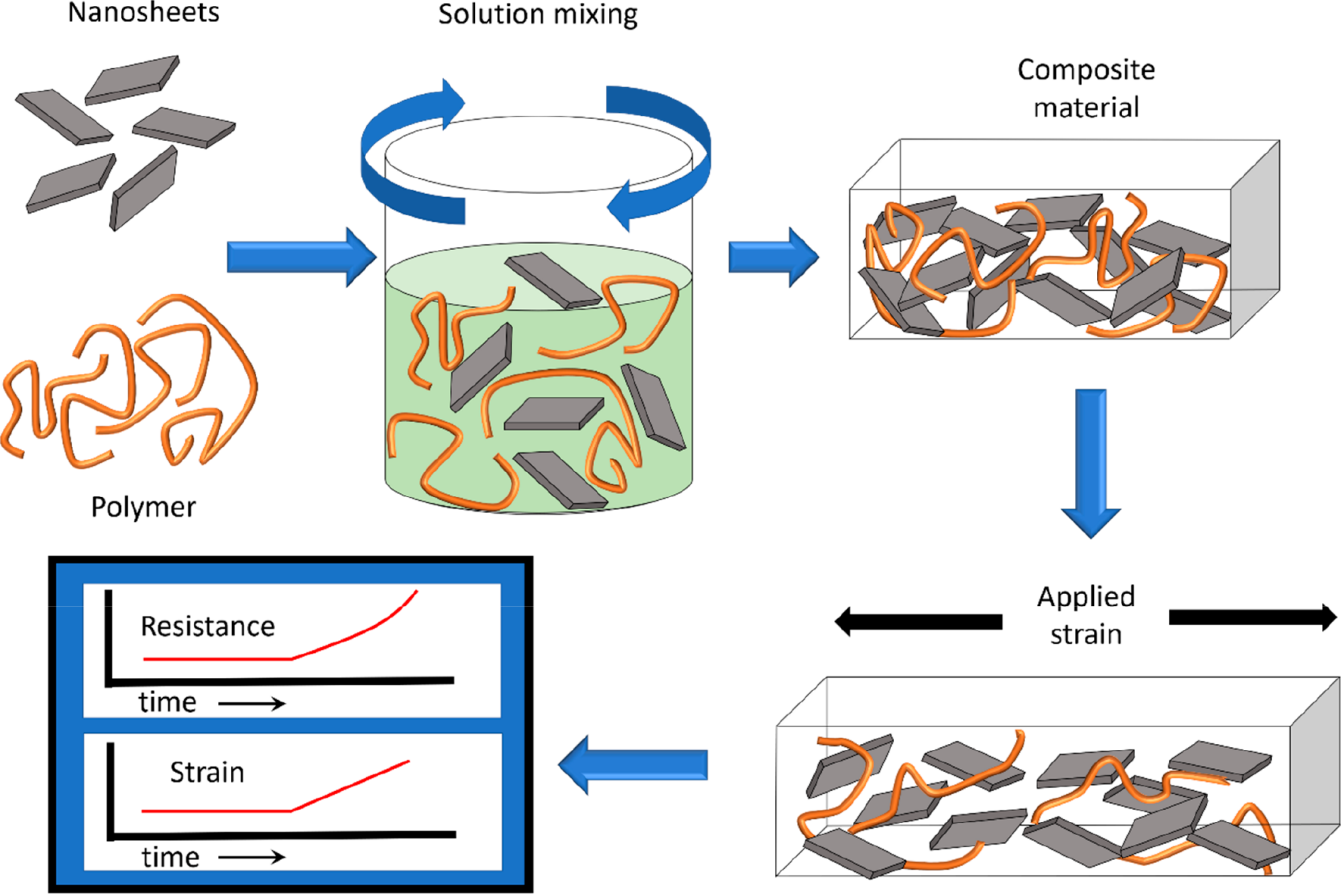
Preparation of Graphene–Polymer Composites Nanosheets and polymer are mixed in solution to yield composite suspensions. The solvent can be removed to yield a polymer–nanosheet composite. Applying strain to this composite deforms the nanosheet network, resulting in a change in its resistance.

**Figure 2:**
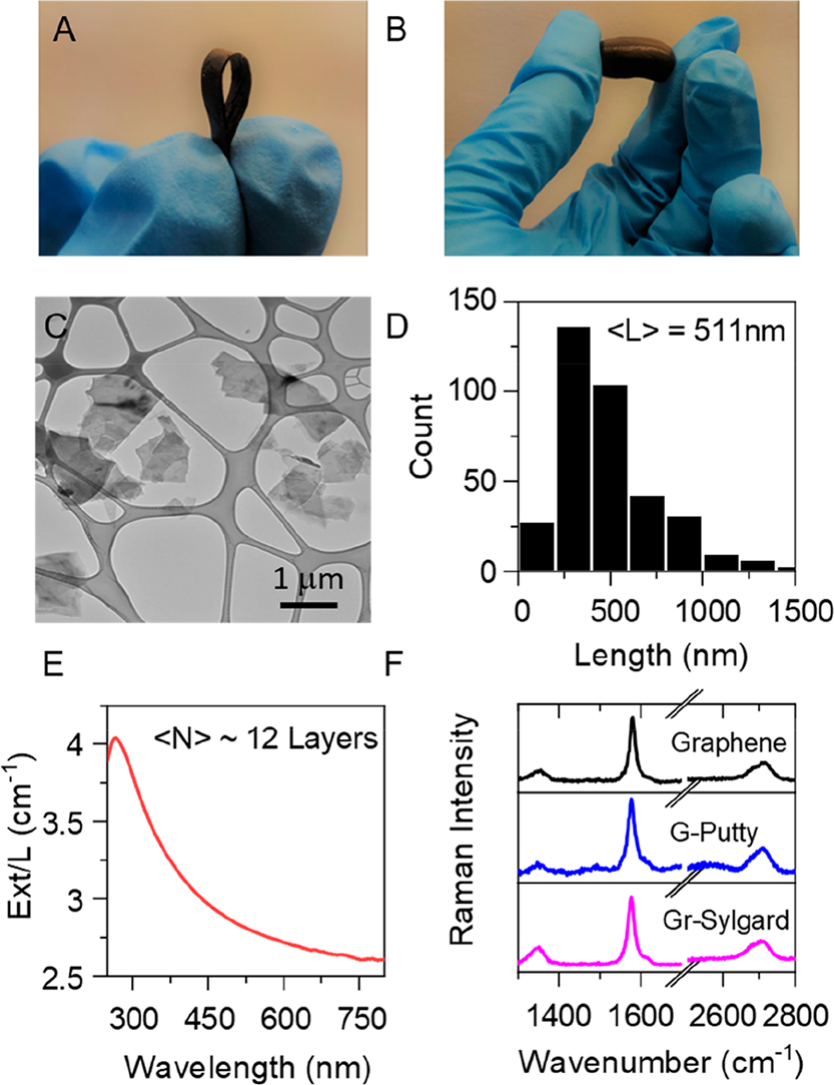
Characterization of G-putty and graphene–sylgard composites: (A, B) Photograph of graphene–sylgard (A) and G-putty (B) composites. (C) TEM images of graphene flakes used in composite fabrication. (D) Histogram of nanosheet length ⟨*L*⟩ = 511 nm acquired through analysis of 368 nanosheets. (E) UV–vis extinction spectra of a graphene dispersion used to make composites with nanosheet thickness estimated using published metrics: ⟨*N*⟩ ∼ 12 layers. (F) Raman spectra for graphene nanosheets as well as G-putty and graphene–sylgard composites.

To test our models, we have prepared graphene–polymer composites using two siloxane-based polymer matrices (see Scheme 1 and Methods). One matrix was the commercially available sylgard polymer which was chemically cross-linked by curing (Figure 2A). The other was a homemade material made by mixing silicone oil with boric acid to yield a soft polymer similar to silly putty, which when mixed with graphene results in a composite known as G-putty (Figure 2B).^15,56^ To achieve this, graphene nanosheets (Figure 2C) were prepared via liquid phase exfoliation with a typical lateral size of ∼511 nm (Figure 2D) and an average layer thickness, ⟨*N*⟩, = 12 layers (Figure 2E), determined from UV–vis measurements using previously published metrics.^57^ Raman spectra for graphene nanosheets as well as G-putty and graphene–sylgard composites consist of the D, G, and 2D bands as expected. The low intensity of the D band indicates the graphene to be defect free while the shape of the 2D band is consistent with few-layer graphene.^57^ In each case, we prepared composites for a range of graphene volume fractions before measuring the zero-strain conductivity (σ_0_) and the gauge factor (*G*) via resistance–strain measurements, each at a range of graphene volume fractions (see the Supporting Information for electromechanical characterization).

**Figure 3:**
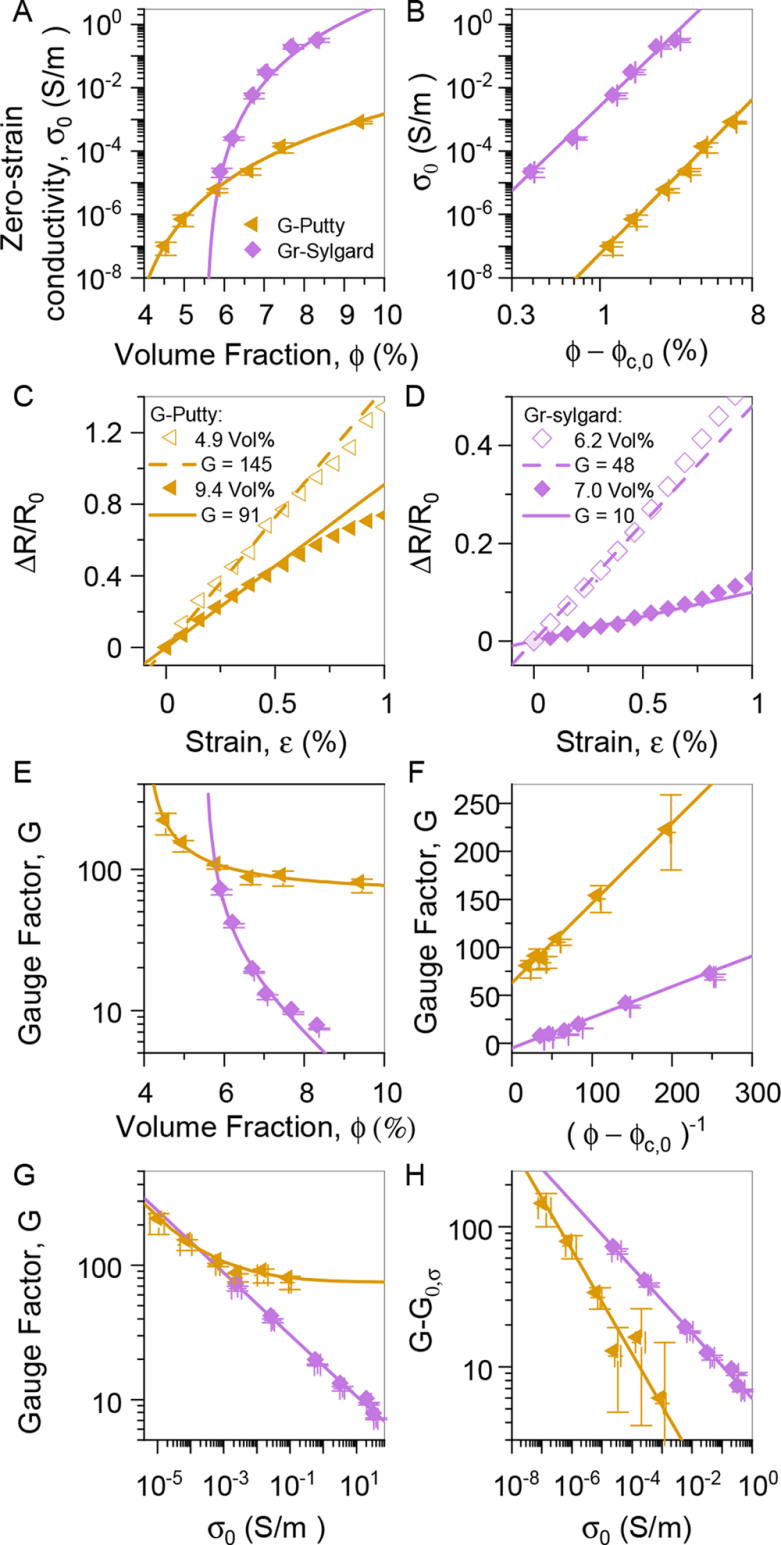
Electromechanical data for polymer–graphene composites prepared and tested during this work. The composites were prepared as described in the text using two different polysiloxane-based polymers filled with liquid-exfoliated graphene. (A, B) Zero-strain conductivity plotted as a function of (A) graphene volume fraction, ϕ, and (B) zero-strain reduced volume fraction, ϕ – ϕ_c,0_. The solid lines are fits to the percolation scaling law, eq 1. G-putty: (ln(σ_c__,0_) = 8.1, ϕ_c__,0_ = 3.4%, and *t*_0_ = 5.4); graphene–sylgard: (ln(σ_c__,0_) = 17.6, ϕ_c__,0_ = 5.5%, and *t*_0_ = 5.1). (C, D) Fractional resistance change plotted versus strain for two different graphene-loading levels for the G-putty composites (C) and graphene–sylgard composites (D). (E, F) Gauge factor plotted versus (E) graphene volume fraction, ϕ, and (F) inverse of zero-strain reduced volume fraction, (ϕ – ϕ_c,0_)^−1^. The solid lines are fits to eq 5a. G-putty: (ϕ_c__,0_ = 4.0%, *G*_1_ = 0.83, *G*_0,ϕ_ = 63); graphene–syglard: (ϕ_c__,0_ = 5.5%, *G*_1_ = 0.32, *G*_0,ϕ_ = −5). (G, H) Gauge factor versus conductivity data plotted as *G* vs σ_0_ (G) and *G* – *G*_0,σ_ vs σ_0_ (H). The solid line is a fit to eq 5b. G-putty: (*t*_0_ = 2.7, σ_1_ = 0.084 S/m, *G*_0*,σ*_ = 75); graphene–syglard: (*t*_0_ = 4.3, σ_1_ = 2220 S/m, *G*_0*,σ*_ = 0.5).

Shown in Figure 3A are values of σ_0_ plotted versus ϕ for both composite types. The solid lines represent fits to eq 1 (see Table 1 for fit parameters). In addition, Figure 3B shows a linearized version of Figure 3A, illustrating the excellent agreement between the data and the model. In both cases, the fit parameters are consistent with previous results. Here we obtained ϕ_c__,0_ = 3.4 vol % and ϕ_c__,0_ = 5.5 vol % for G-putty and graphene sylgard composites. While these percolation thresholds are relatively high, a review of graphene–polymer composites by Marsden et al.^17^ demonstrates that a broad range of percolation threshold values exists, 0.004–5 vol %, for nanocomposites with graphene and other carbon-based fillers. Additionally, Wang et al.^53^ report a similar graphene–siloxane-based sensor with ϕ_c__,0_ = 8.1 vol %.

**Table 1:** Various Parameters Obtained from Linearized Fits of Data to Eqs 1 (σ_0_ vs ϕ), 5a (*G* vs ϕ), and 5b (*G* vs σ_0_)^a^

	G-sylgard	G-putty
From Fitting σ_0_ vs ϕ		
ln(σ_c_,_0_)	17.6 ± 1.1	8.1 ± 0.6
ϕ_c__,0_	0.055 ± 0.001	0.034 ± 0.001
*t*_0_	5.1 ± 0.2	5.4 ± 0.3
From Fitting *G* vs ϕ		
*G*_0,ϕ_	–5 ± 1.4	63 ± 3
*G*_1_	0.32 ± 0.01	0.83 ± 0.03
ϕ_c__,0_	0.055 ± 0.00	0.040 ± 0.001
From Fitting *G* vs σ_0_		
*G*_0,σ_	0.5 ± 0.1	75 ± 5
σ_1_ (S/m)	2220 ± 110	0.084 ± 0.042
*t*_0_	4.3 ± 0.2	2.7 ± 0.3

a Corresponding plots are shown in Figure 3B, F, and H.

Values of ln(σ_c__,0_) = 8.1, 17.6 were obtained for G-putty and graphene–sylgard composites. Literature values for σ_c_ vary over many orders of magnitude. For example, epoxy–graphene composites have been reported with values as low as ln(σ_c,0_) ∼ −13.8,^58^ whereas polystyrene–graphene composites have been shown to give ln(σ_c__,0_) ∼ 11.5.^54^ The values reported here lie at the upper end of the literature.

The percolation exponents were 5.4 and 5.1 for G-putty and graphene sylgard composites. We note that the percolation exponents are both somewhat higher than the universal value of *t*_un_ = 2 in 3D. This is very common^29^ for polymer-based nanocomposites with higher values of *t* indicating the presence of a broad distribution of internanosheet junction resistances.^44−46^

As is almost^28^ always observed, the nanocomposite resistance increased with strain (Figure 3C–D), a fact that is usually attributed to the effect of strain on interparticle junctions.^19,25,31^ In all cases, the fractional resistance change scaled linearly with strain at low strain with some nonlinearity appearing at strains above ∼0.75%. In these curves the low-strain slope is equal to the gauge factor which has been extracted for a range of volume fractions for each composite type.

The resultant gauge factors are plotted versus ϕ in Figure 3E for both composite types. We have fit both data sets using eq 5a, finding very good matches (see Table 1 for fit parameters). In both cases, the data (and fit) diverge as ϕ → ϕ_c__,0_ from above, in line with the prediction of eq 5a. In order to best assess the agreement between the data and the model, we plot the data in a manner which, according to the model, should yield a straight line (Figure 3F). This does indeed yield a straight line, confirming that the model describes the data very well and that the approach of treating the ln term in eqs 4b–4c is a valid one. In addition, these fits introduce a somewhat unexpected fact, that the *G*_0,ϕ_ parameter (represented by the intercept in Figure 3F) can be negative. This will be discussed in more detail below.

As shown in Figure 3G, we have also plotted the gauge factor versus the zero-strain composite conductivity (reproduced from Figure 1). We have fitted both data sets to eq 5b, again getting good agreement (see Table 1 for fit parameters). In addition, we demonstrate the fidelity of the model to the data by plotting the data in a linearized fashion in Figure 3H, again finding very good agreement.

### Fit Parameters

As described above, standard strain sensor measurements lead to three distinct data sets (σ_0_ vs φ, *G* vs φ, and *G* vs σ_0_) that can be fit using eqs 1, 5a, and 5b, yielding nine fit parameters as listed in Table 1. It is clear from Table 1 that there is some degree of redundancy in these fit parameters. For example, the percolation threshold (ϕ_c__,0_) can be extracted from fitting both σ_0_ vs ϕ and *G* vs ϕ data while the percolation exponent (*t*_0_) can be extracted from fitting σ_0_ vs ϕ and *G* vs σ_0_ data sets. In addition, inspection of eqs 4b and 4c, in combination with eq 1, shows that the parameters *G*_0,ϕ_ and *G*_0,σ_ are actually identical. Thus, under normal circumstances, it would not be necessary to fit all three data sets, with most researchers probably opting to fit only σ_0_ vs ϕ and *G* vs ϕ.

While the percolation fit parameters (σ_c__,0_, ϕ_c__,0_, and *t*_0_) are well-known, obviously the parameters *G*_0,ϕ_, *G*_1_, *G*_0,σ_, and σ_1_ will be new to readers. It is important to assess the range of values these parameters can take in composites. To achieve this, we have used eqs 5a–5b to fit the published data sets shown in Figure 1 (see the Supporting Information). This analysis shows that *G*_0,ϕ_ and *G*_0,σ_ can both display values between −200 and 80. In addition, we find the minimum ranges of *G*_1_ and σ_1_ to be 10^–2^ < *G*_1_ < 10^2^ and 10^–4^ < σ_1_ < 10^13^ (see the Supporting Information).

### Rate of Change of Percolation Parameters with Strain

**Figure 4:**
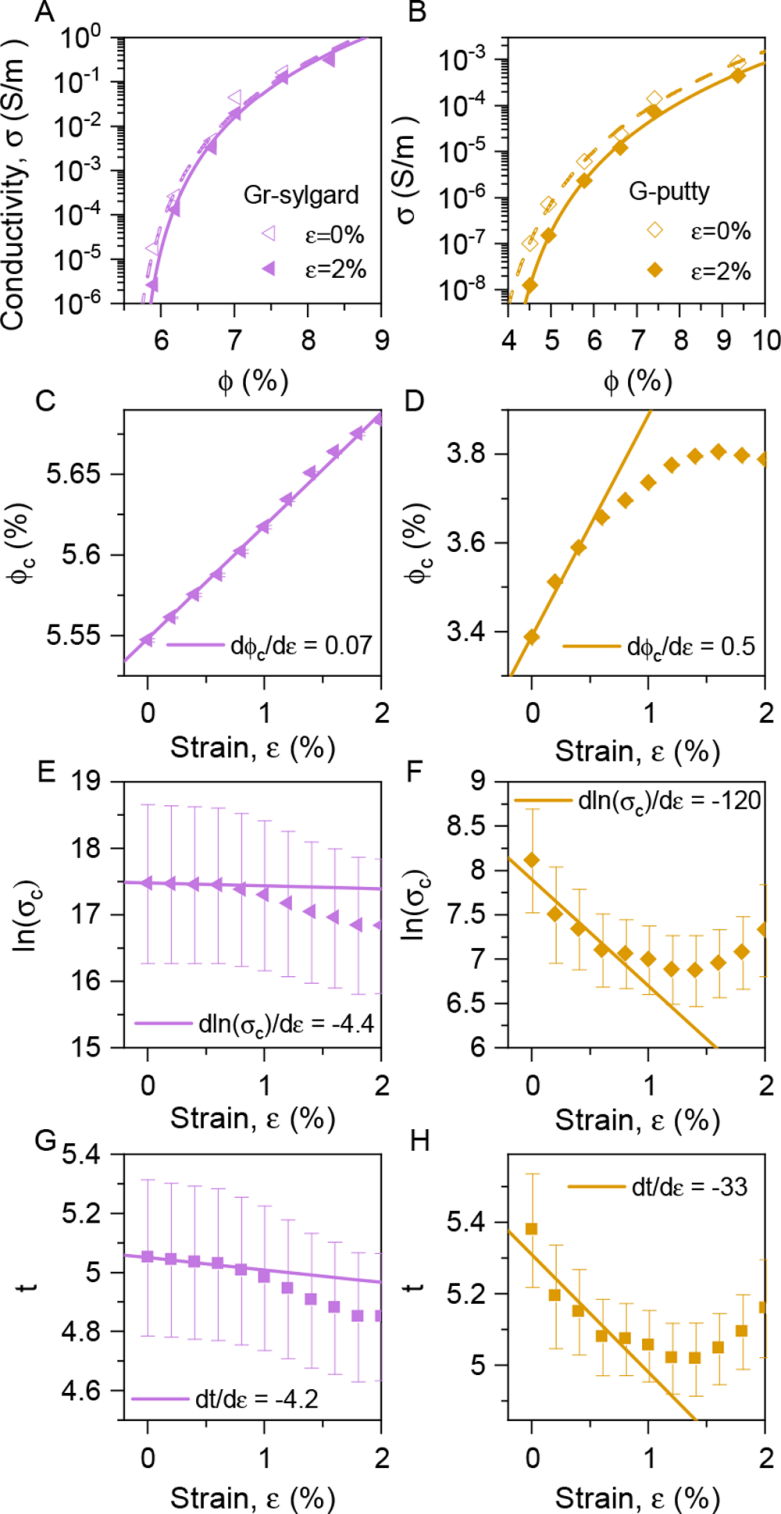
Strain-dependent percolation data. (A–B) Composite conductivity plotted versus graphene volume fraction for the G-putty composites (A) and graphene–sylgard composites (B). The lines are fits to the percolation scaling law (eq 1) which outputs the fit parameters σ_c,0_, ϕ_c,0_, and *t*_0_. For both composites, such fits were obtained for a range of strain values. (C–H) Plots of percolation parameters, percolation threshold, ϕ_c_ (C–D), ln(σ_c_) (E–F) and percolation exponent, *t* (G–H), all plotted versus strain for both composite types (graphene–sylgard in the left column, G-putty in right the column). The lines represent linear fits that give the slope of the curves at low strain.

The analysis above shows eqs 5a–5b to accurately represent the experimental data. However, it would be ultimately more useful to determine how accurately eqs 4b–4c match the experimental data, as it is these equations which contain the physics describing the piezoresistive process. As we already have data for σ_c__,0_, ϕ_c__,0_, and *t*_0_, testing these equations requires knowledge of (dln σ_c_/dε)_0_, (dϕ_c_/dε)_0_, and (d*t*/dε)_0_. To obtain these parameters, we first take the resistance versus strain measurements (at 0.2% strain increments) recorded for various volume fractions (e.g, Figure 3C–D) and convert them to resistance versus volume fraction data sets, each at various strains (limiting ourselves to strains from 0 to 2%). For each strain, the set of resistances were converted to conductivity assuming constant sample volume:
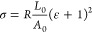
6where *L*_0_ and *A*_0_ are the zero-strain sample length and cross-sectional area. This procedure yields a set of conductivity versus volume fraction data sets, each at a different strain. Examples of such curves, associated with strains of 0% and 2%, are shown in Figure 4A–B. These curves clearly show slight reductions in conductivity with strain at all volume fractions. These curves were then fit to the percolation scaling law (eq 1) yielding values of ln σ_c_, ϕ_c_, and *t* as a function of strain for each composite as plotted in Figure 4C–H.

As shown in both Figure 4C and D, both composites show a clear increase of percolation threshold with strain, leading to low-strain slopes, (dϕ_c_/dε)_0_, of 0.07 and 0.5 for graphene–sylgard and G-putty composites, respectively. These results agree with previous studies which showed increases in percolation threshold both with strain^32^ and under alignment.^59^ For example, Zhang et al. measured (dϕ_c_/dε)_0_ = 0.004 for polyurethane–nanotube composites.^32^ Such positive slopes are to be expected as described above.

As shown in Figure 4E–F, both composites show reductions in ln σ_c_ with increasing strain, although for the graphene–sylgard composites the change is small compared to the error bars. Notwithstanding the error, the low-strain values of (dln σ_c_/dε)_0_ are −4.4 and −120 for graphene–sylgard and G-putty composites, respectively. This can be compared with values of (dln σ_c_/dε ≈ −30, which can be extracted from ref (32). As described above, we expect these slopes to be negative.

The values of dln σ_c_/dε = −*kd*_0_ are different by a factor of 27 between the graphene–sylgard and G-putty composites. If a nontrivial amount of interparticle charge transport is to occur, the value of *d*_0_ must lie in a relatively narrow range (*d*_0_ can never be less than the van der Waals distance while values greater than a few nanometers will result in negligible tunnelling current). This means that much of this large difference is probably associated with variations in *k* between composites. As *k* is presumably controlled by the details of the interparticle potential barrier, this shows that the nature of the junction can have a significant impact on the gauge factor.

Both composites also show reductions in *t* with increasing strain (Figure 4G–H). However, as before, the graphene–sylgard composites show a small change compared to the size of the error bars. The low-strain values of (d*t*/dε)_0_ are −4.2 and −33 for graphene–sylgard and G-putty composites, respectively. Both of these results show a reduction in *t* with strain, consistent with the only other published work we could find on this topic.^32^ Zhang et al. investigated electromechanical properties of MWCNT–polyurethane composites, finding (d*t*/dε)_0_ = −8.^32^ We note that this value has the same sign as (dln σ_c_/dε)_0_, as indicated above.

### Using These Derivatives to Model the Gauge Factor

**Figure 5:**
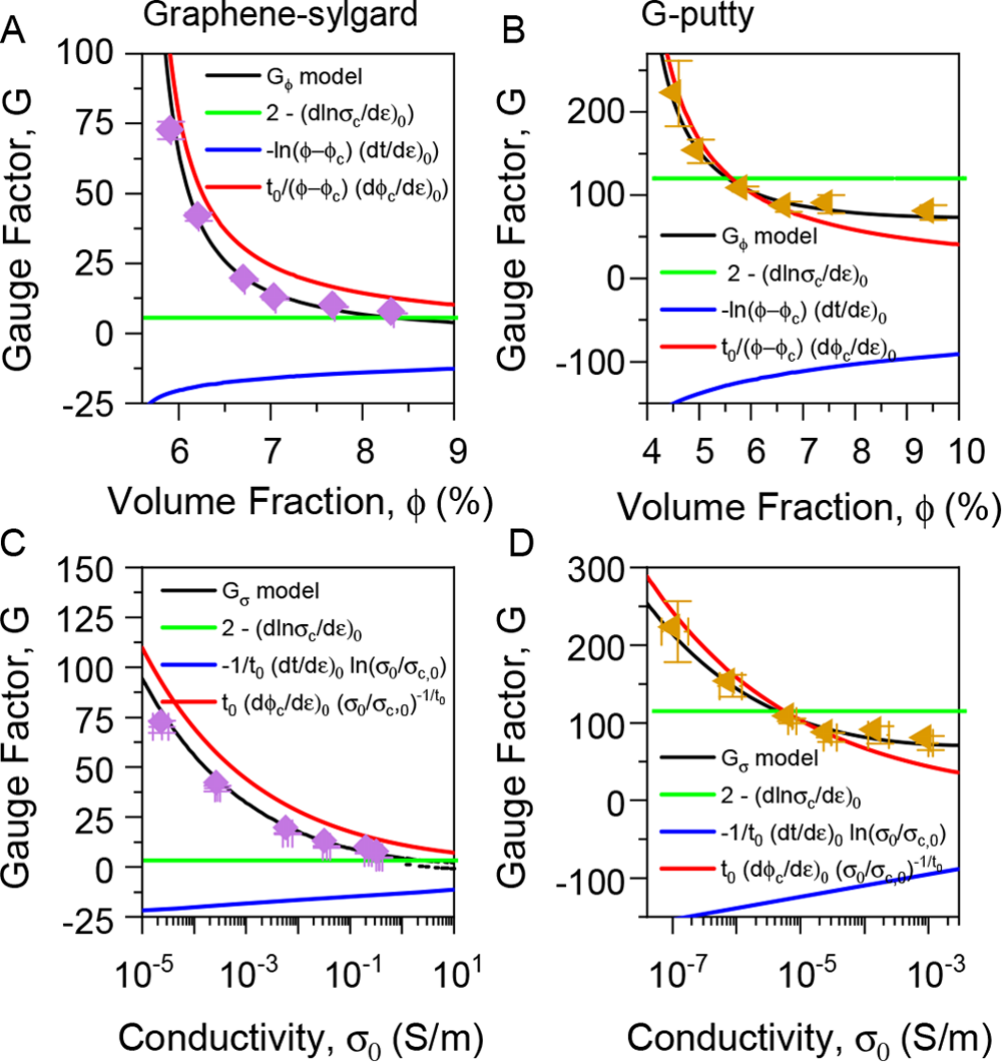
Plotting model predictions. Experimental gauge factor data plotted versus graphene volume fraction (A–B) and zero-strain conductivity (C–D) for graphene–sylgard (A, C) and G-putty (B, D) composites. In each panel, the black lines are obtained by plotting either eq 5b (A–B) or 5a (C–D) using the parameters obtained in Figure 4. In each panel, the green, blue, and red lines represent the first, second, and third terms respectively in eqs 5a and 5b.

Once values for all of the quantities in eqs 4b and 4c are known, these equations can be used to plot numerical graphs of *G* vs ϕ and *G* vs σ_0_. Comparison with experimental data would provide evidence as to the accuracy of eqs 4b and 4c. Substituting values of σ_c__,0_, ϕ_c__,0_, and *t*_0_ from Table 1 (choosing the values appropriate to each graph and each material), as well as values for (dln σ_c_/dε)_0_, (dϕ_c_/dε)_0_, and (d*t*/dε)_0_ (Table 2), for both graphene–sylgard and G-putty composites into eqs 4b and 4c yields plots of *G* vs ϕ and *G* vs σ_0_ as shown in Figure 5 (black lines). These curves match the experimental data extremely well, illustrating the validity of our approach.

**Table 2:** Parameter Values (Eqs 4a, 4b, and 4c) Obtained from Linear Fits at Low Strain to the Percolation Data Presented in Figure 4C–H

	(dln σ_c_/dε)_0_	(dϕ_c_/dε)_0_	(d*t*/dε)_0_
expected sign	–ve	+ve	–ve
G-sylgard value	–4.4	0.07	–4.2
G-putty value	–120	0.5	–33

This agreement between model prediction and data allows us to assess the magnitude of the contributions of each term in eqs 4b–4c. We use the same parameters as before to plot each term individually on each panel in Figure 5. The first thing to note is that the second term (blue line) depends only weakly on ϕ and σ_0_ justifying our assertion that the ln terms could be treated as constant. In addition, the first (green line) and third (red line) terms are positive while the second term (blue line) is negative as described above. In addition, the first and second terms are similar in magnitude which means that they somewhat cancel each other out. Depending on the degree of cancellation, this can result in relative small or negative values of *G*_0,ϕ_ and *G*_0,σ_, limiting the positive contribution of the first two terms to the gauge factor. Essentially, this means that the third term can be particularly important, especially for volume fractions approaching the percolation threshold.

The fact that eqs 4b and 4c describe the experimental data so well means we should revisit the assertion, made earlier, that these equations should not be used to fit data (we suggested using eqs 5a–5b). Both eqs 4b and 4c have five fit parameters, which is too many to allow reliable fitting of standard data sets. However, it is worth considering if eqs 4b and 4c can be used for fitting data in order to obtain values for dlnσ_c_/dε, dϕ_c_/dε, and d*t*/dε if the percolation fit parameters (σ_c__,0_, ϕ_c__,0_, *t*_0_) were used as fixed values. We attempted to achieve this for the graphene–sylgard and G-putty data sets as shown in the Supporting Information (Figure S9). We achieved mixed results, obtaining some reasonable values of dlnσ_c_/dε, dϕ_c_/dε, and d*t*/dε and some results which were far from the expected values or which had large errors. We believe the main problem here is associated with the limited number of data points per data set (six in this case). Although such data sets are standard or even extensive compared to the literature, they are clearly not enough to reliably extract the derivatives. However, this might be addressed in the future, simply by fabricating larger sample sets with more filler volume fractions, leading to more data points per data set.

### Factors Effecting the Gauge Factor

Now that it is reasonably clear that eqs 4b and 4c can quantitatively describe experimental data, it is worth considering what we require of each parameter in order to maximize *G*. For simplicity, we will focus on eq 4b.

Ideally, we would want the sum of the first two terms to be large and positive. Given its expected negative sign, this means we want |(dln σ_c_/dε)_0_| to be as large as possible to maximize the first term. In addition, because we expect (d*t*/dε)_0_ and so the entire second term to be negative, we need |(d*t*/dε)_0_| to be small while we would like *t*_0_ to be large. However, if, as described above, (dln σ_c_/dε)_0_ ∝ (d*t*/dε)_0_, it is clearly not possible to achieve these conditions simultaneously. If it were possible to engineer the properties of the composite, the most pragmatic strategy might be to maximize |(dln σ_c_/dε)_0_| and *t*_0_ in the hope that |(d*t*/dε)_0_| is not too large to negatively affect *G*. Maximizing |(dln σ_c_/dε)_0_| means maximizing the product *kd*_0_ mentioned above. Clearly, to do this requires an understanding of the interparticle transport mechanism and so *k*. However, if for example the relevant mechanism is Simmon’s tunnelling as assumed in refs (19 and 30), then the maximization of *k* requires the maximization of the height of the interparticle tunnelling barrier. This might be achievable by coating the conducting particles with a wide-bandgap insulator.

**Figure 6:**
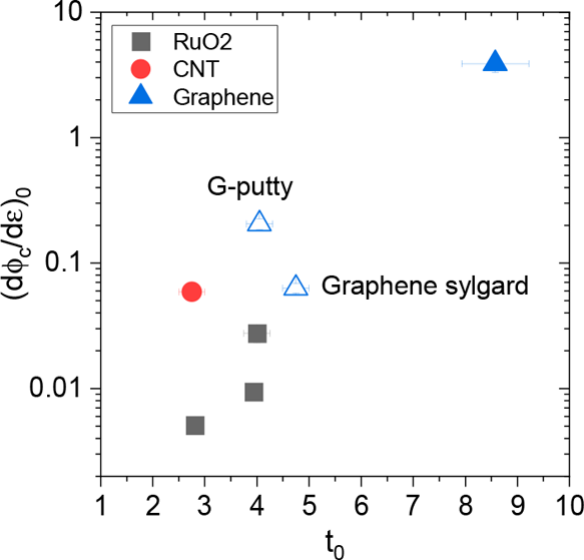
Values of (dϕ_c_/dε)_0_ plotted versus *t*_0_ for the literature data reported in Figure 1 (solid symbols, see the Supporting Information for fits) as well as the samples prepared in this work (open symbols). The values for *t*_0_ are the averages of the values found by fitting the σ_0_ vs ϕ and *G* vs σ_0_ data sets.

The third term in eq 4b is less ambiguous. This term will always be positive and will be maximized for large values of *t*_0_ and (dϕ_c_/dε)_0_. As mentioned above. *t*_0_ is associated with the width of the distribution of interparticle junction resistances, a parameter that might somehow be engineered. The nature of (dϕ_c_/dε)_0_ is less clear. However, we note that if *t*_0_ is known (for example, by fitting conductivity data), (dϕ_c_/dε)_0_ can be obtained from *G*_1_ (obtained by fitting *G* vs φ data to eq 5a). To shed more light on what values of (dϕ_c_/dε)_0_ are possible, we estimated (dϕ_c_/dε)_0_ by fitting the literature data shown in Figure 1 (see the Supporting Information) to obtain *G*_1_. These values were combined with the *t*_0_ values from Figure S8B (here we took the average of both *t*_0_ values for each material) to obtain (dϕ_c_/dε)_0_. To illustrate these values, we plot the resultant values of (dϕ_c_/dε)_0_ versus *t*_0_ as shown in Figure 6. We find a well-defined relationship with larger values of *t*_0_ leading to larger values of (dϕ_c_/dε)_0_.

We can understand this relationship as follows. Large values of *t*_0_ indicate a broad distribution of interparticle junction resistances. Consider a composite with large *t*_0_ very close to the percolation threshold. Under these circumstances, only one complete current path exists which will have at least one bottleneck. The larger the value of *t*_0_, the larger the probability that the limiting interparticle junction is one of high resistance. Because high-resistance junctions are likely to be associated with large interparticle separation (*R*_J_ ∝ e^*kd*^), they are more likely to be broken under strain. Such breakage will destroy the current path, shifting the percolation threshold (at that strain) to higher filler volume fraction. We expect such circumstances to lead to high (dϕ_c_/dε)_0_, illustrating the link between this parameter and *t*_0_.

The discussion above illustrates the importance of *t*_0_. This is probably the most important parameter of all due to its double influence on the third term in eq 4b as well as its role in reducing the magnitude of the (negative) second term. We believe that the maximization of *G* would be significantly enhanced if it became possible to find ways to engineer composites to have high values of *t*_0_.

## Conclusion

In conclusion, we have used percolation theory to develop a model relating the nanocomposite gauge factor (sensitivity) to the filler volume fraction in piezoresistive sensors. This model predicts the gauge factor to diverge as the filler volume fraction approaches the percolation threshold from above, a key feature observed experimentally for nanocomposite sensors. In addition, alongside the widely considered contribution from the interparticle resistance, the model shows the gauge factor to depend strongly on effects associated with the network of filler particles.

The model is in good agreement with experimental data, both measured here and extracted from the literature. In addition, once the percolation fit parameters and their strain derivatives were independently obtained from experimental data and inserted into the model, gauge factors could be predicted to a good degree of accuracy.

These results are important for two reasons. First, our equations can be used to fit the experimental data, yielding figures of merit for piezoresistive performance. This allows the comparison of strain sensors both with each other and with the literature. More importantly, this work shows the response of composite strain sensors to be more complex than previously thought and shows the effect of strain on the particle network to be at least as important as the effect of strain on the interparticle resistance.

## Methods

### Graphene

A graphene dispersion was prepared by ultrasonic tip sonication (Hielscher UP200S, 200 W, 24 kHz) of graphite (Branwell, graphite grade RFL 99.5) in 1-methyl-2-pyrrolidone (NMP) (Sigma-Aldrich, HPLC grade) at a concentration of 100 mg/mL for 72 h at an amplitude of 60%. The resulting dispersion then underwent mild centrifugation at 1500 rpm for 90 min to remove unexfoliated aggregates and large nanosheets. The supernatant was then vacuum filtered on a 0.1μm nylon membrane, forming a thick disk of reaggregated graphene nanosheets. This disk was then ground into a fine powder using a mortar and pestle before being added to separate solutions of chloroform (10 mg/mL) and IPA (1 mg/mL). Graphene was then redispersed in each solvent by tip sonication for 90 min at 40% amplitude to form stock solutions.

### G-Putty

G-putty is a viscoelastic siloxane-based graphene composite described in previous works.^15,56^

First, silicone oil was partially cross-linked using boric acid which resulted in the formation of a material similar to silly putty. Two milliliters of silicone oil (VWR CAS No.: 63148-62-9, 350 cSt) was added to a 28 mL glass vial. Boric acid (Sigma-Aldrich 99.999% trace metal basis, CAS: 10043-35-3) was ground with a mortar and pestle until a fine powder was formed. This powder was then added to the silicone oil at a concentration of 0.4 g/mL and stirred by magnetic stirrer bars until the mixture was homogeneous and opaque. The glass vials were then added six at a time to a specially prepared aluminum holder.

An oil bath was preheated to 175 °C (IKA C-MAG HS 7 hot plate with the temperature controlled and monitored using an ETS-D5 thermometer), the aluminum holder was then submerged in the bath, and the temperature was increased to 225 °C. Silicone oil/boric acid mixture was cured for ∼2.5 h (including heating time from 175 to 225 °C) under continuous magnetic stirring. The vials were then removed from the oil bath and allowed to cool. Once cooled, the resulting material was a viscoelastic gum, which could be removed from the vials with a spatula.

A 0.5 g sample of the viscoelastic gum was added to a beaker with the appropriate amount of graphene–chloroform solution, depending on the required graphene loading. Under magnetic stirring the mixture was heated to 40 °C, and the solvent was allowed to evaporate until a thick black viscous liquid had formed. The beaker was then removed from the heat and left to stand for 12 h to ensure that all the solvent had evaporated. The resulting composite was removed from the beaker and repeatedly folded over itself to ensure the homogeneity of the sample.

### Graphene–Sylgard

A 0.4 g sample of Sylgard 170 (Dow Corning) Part A and Part B was added to a beaker containing 10 mL of the graphene–IPA dispersion and stirred under magnetic stirring for 2 min. Further graphene–IPA was then added depending on the required graphene loading. The mixture was gently heated to 40 °C, and the solvent was allowed to evaporate under continuous stirring. Once almost all of the solvent had evaporated, the mixture was transferred into Teflon molds (35 × 35 mm). The mixture was left to stand for 12 h to ensure complete solvent evaporation and then cured at 100 °C for 1 h in an oven. The final composite was removed from the mold and measured ∼600 μm in thickness. Details on composite characterization can be found in the Supporting Information

## Supporting Information Available

Composite characterization methods; electromechanical composite characterization; extracted fit parameters and fitted data from literature sources (PDF)
