# Supplementary material for: A Simple Model Relating Gauge Factor to Filler Loading in Nanocomposite Strain Sensors
Source: ACS Appl Nano Mater. 2021 Mar 5;4(3):2876–86. doi: 10.1021/acsanm.1c00040 (PMC8862007; doi:10.1021/acsanm.1c00040)
Supplement: Supplementary file 1 — an1c00040_si_001.pdf [file an1c00040_si_001.pdf]

SUPPORTING INFORMATION:

A Simple Model Relating Gauge Factor to Filler Loading in Nanocomposite Strain Sensors

James R. Garcia[1], Domhnall O'Suilleabhain[1], Harneet Kaur and Jonathan N. Coleman[1]*

[1]*School of Physics, CRANN & AMBER Research Centres, Trinity College Dublin, Dublin 2, Ireland*

*colemaj@tcd.ie (Jonathan N. Coleman); Tel: +353 (0) 1 8963859.

Methods: Composite Characterisation

Resistance measurements were carried out using a Keithley KE2601 source meter in a 2-probe mode, controlled by LabView software. Electromechanical tests are carried out in conjunction with a Zwick Z0.5 ProLine Tensile Tester (100 N Load Cell). Graphene-sylgard samples (35 ×35mm) where cut into strips, 3.5mm in width, while viscoelastic G-putty samples where moulded into a cylindrical shape ~1mm. All samples had a gauge length, L_0 = 9mm. Tensile measurements were carried out at a strain rate of 1.85%/s. At each filler volume fraction a total of 6 resistance-strain were carried out and an average value of G was obtained.

Raman spectra were acquired with Horiba Jobin Yvon set up using a laser line of 532 nm. A 10X objective was used with a neutral density filter of 25 to avoid heating of sample.

A JEOL JEM-2100 LaB6 transmission electron microscope operating at 200 kV was used to provide images of individual nanosheets. The dispersion was diluted to low concentrations and drop-cast onto an ultrathin carbon film TEM grid provided by Ted Pella, Inc. The TEM grid was placed on a piece of filter paper in order to wick away any excess solvent and then dried overnight in a vacuum oven. The images were used to obtain a statistical analysis of the nanosheet lengths, here defined as the longest axis of each nanosheet.

UV–visible spectroscopy was performed using a Perkin Elmer UV-Vis Spectrophotometer to optically characterise the exfoliated nanosheet dispersion. The wavelength was scanned

between 800 and 200 nm, and a 4 mm path length-reduced volume quartz cuvette was used for the measurement.

Gauge factor derivation

The definition of the gauge factor:

$$\frac{\Delta R}{R_0} = G\varepsilon \qquad \text{(S.1)}$$

The resistance of a sample can be related to its conductivity by:

$$R = \frac{L}{\sigma A} \qquad \text{(S.2)}$$

Assuming that the volume remains constant at low strain, $AL = A_0 L_0$, then equation S.2 can be re-written as :

$$R = \frac{L^2}{\sigma A_0 L_0} \qquad \text{(S.3)}$$

Differentiating R with respect to ε yields:

$$\frac{dR}{d\varepsilon} = \frac{1}{A_0 L_0}\left[2L\frac{1}{\sigma}\frac{dL}{d\varepsilon} - \frac{L^2}{\sigma^2}\frac{d\sigma}{d\varepsilon}\right] \qquad \text{(S.4)}$$

Now divide through by the zero-strain resistance $R_0 = L_0 / A_0\sigma_0$:

$$\frac{1}{R_0}\frac{dR}{d\varepsilon} = \frac{\sigma_0}{L_0^{\;2}}\left[2L\frac{1}{\sigma}\frac{dL}{d\varepsilon} - \frac{L^2}{\sigma^2}\frac{d\sigma}{d\varepsilon}\right] \qquad \text{(S.5)}$$

At low strain, $L \approx L_0$ and $\sigma \approx \sigma_0$, so Equation S.5 becomes:

$$\frac{1}{R_0}\frac{dR}{d\varepsilon} = \left[2\frac{dL / L_0}{d\varepsilon} - \frac{1}{\sigma_0}\frac{d\sigma}{d\varepsilon}\right] \qquad \text{(S.6)}$$

Since the definition of strain is simply $\varepsilon = (L - L_0) / L_0$, we finally obtain:

$$\frac{1}{R_0}\frac{dR}{d\varepsilon} = G \approx 2 - \frac{1}{\sigma_0}\frac{d\sigma}{d\varepsilon} \qquad \text{(S.7)}$$

Model derivation

In classical percolation theory, composite conductivity, σ is related to the filler volume fraction, ϕ, by[1, 2] :

$$\sigma = \sigma_c(\phi - \phi_c)^t \qquad \text{(S.8)}$$

Consider that $\sigma_c = \sigma_c(\varepsilon)$, $\phi_c = \phi_c(\varepsilon)$ and $t = t(\varepsilon)$, since we are only concerned with the low strain region where the volume of the sample is assumed constant, so too is the volume of the filler, ϕ , assumed to be invariant with strain. Substituting equation s8 into equation s7 will yield an equation for G in terms of the percolation parameters.

Firstly consider $d\sigma / d\varepsilon$:

$$\frac{d\sigma}{d\varepsilon} = (\phi - \phi_c)^t \frac{d\sigma_c}{d\varepsilon} + \sigma_c \frac{d}{d\varepsilon}(\phi - \phi_c)^t \qquad \text{(S.9)}$$

Dealing with the second part of this equation:

$$\frac{d}{d\varepsilon}(\phi - \phi_c)^t = (\phi - \phi_c)^t \frac{d}{d\varepsilon}[t \ln(\phi - \phi_c)] \qquad \text{(S.10)}$$

$$\frac{d}{d\varepsilon}[t \ln(\phi - \phi_c)] = \ln(\phi - \phi_c)\frac{dt}{d\varepsilon} + t\frac{d}{d\varepsilon}\ln(\phi - \phi_c) \qquad \text{(S.11)}$$

$$\frac{d}{d\varepsilon}\ln(\phi - \phi_c) = -\frac{d\phi_c / d\varepsilon}{\phi - \phi_c} \qquad \text{(S.12)}$$

Therefore we can write:

$$\frac{d\sigma}{d\varepsilon} = \sigma_c\left(\phi - \phi_c\right)^t\left(\frac{1}{\sigma_c}\frac{d\sigma_c}{d\varepsilon} + \ln(\phi - \phi_c)\frac{dt}{d\varepsilon} - \frac{t}{\phi - \phi_c}\frac{d\phi_c}{d\varepsilon}\right) \qquad \text{(S.13)}$$

Substituting Equation S.13 into Equation S.7:

$$G \approx 2 - \frac{1}{\sigma_0}\left[\sigma_c\left(\phi - \phi_c\right)^t\left(\frac{1}{\sigma_c}\frac{d\sigma_c}{d\varepsilon} + \ln(\phi - \phi_c)\frac{dt}{d\varepsilon} - \frac{t}{\phi - \phi_c}\frac{d\phi_c}{d\varepsilon}\right)\right] \qquad \text{(S.14)}$$

Literature data

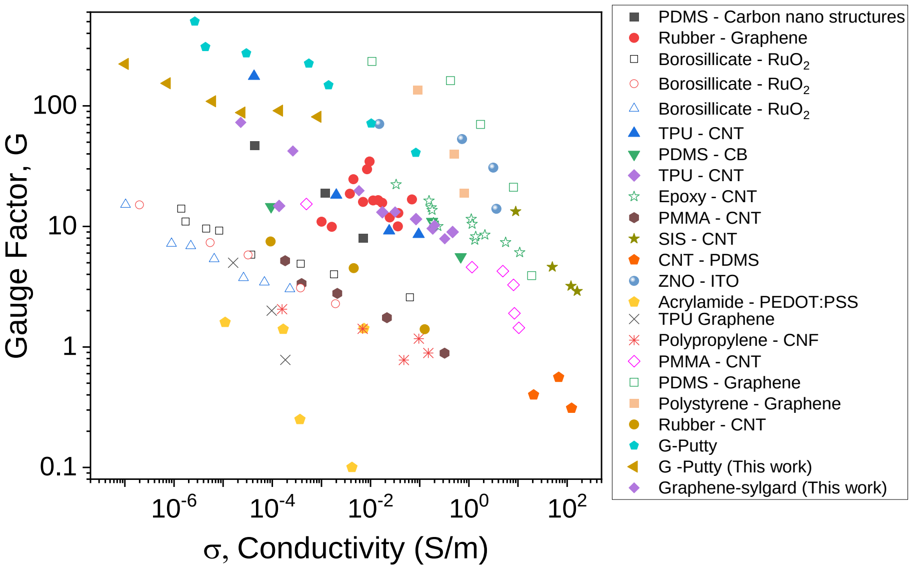

Figure S1: Literature data[3-20] for Gauge factor (G) as a function of zero-strain conductivity (σ_0) for a range of nanocomposites. Here we observe that for almost all composites G decreases as conductivity increases. A look at the data indicates an approximate power law relationship between these parameters for well-defined data sets.

Composite Characterisation

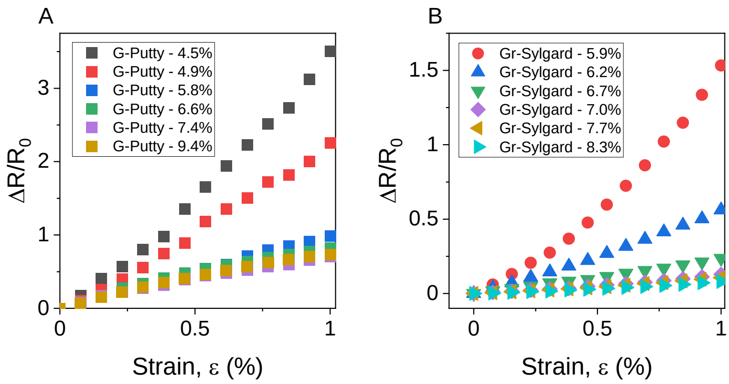

Figure S2: Electro-mechanical characterisation: Representative resistance-strain measurements for G-putty (A) and Graphene-sylgard (B). As graphene volume fraction approaches the percolation threshold composite resistance becomes increasingly sensitive to strain, this leads to larger Gauge factor, G values, which can be obtained by linear fits to the data as in Figure 3C,D (main text).

Fits to literature data

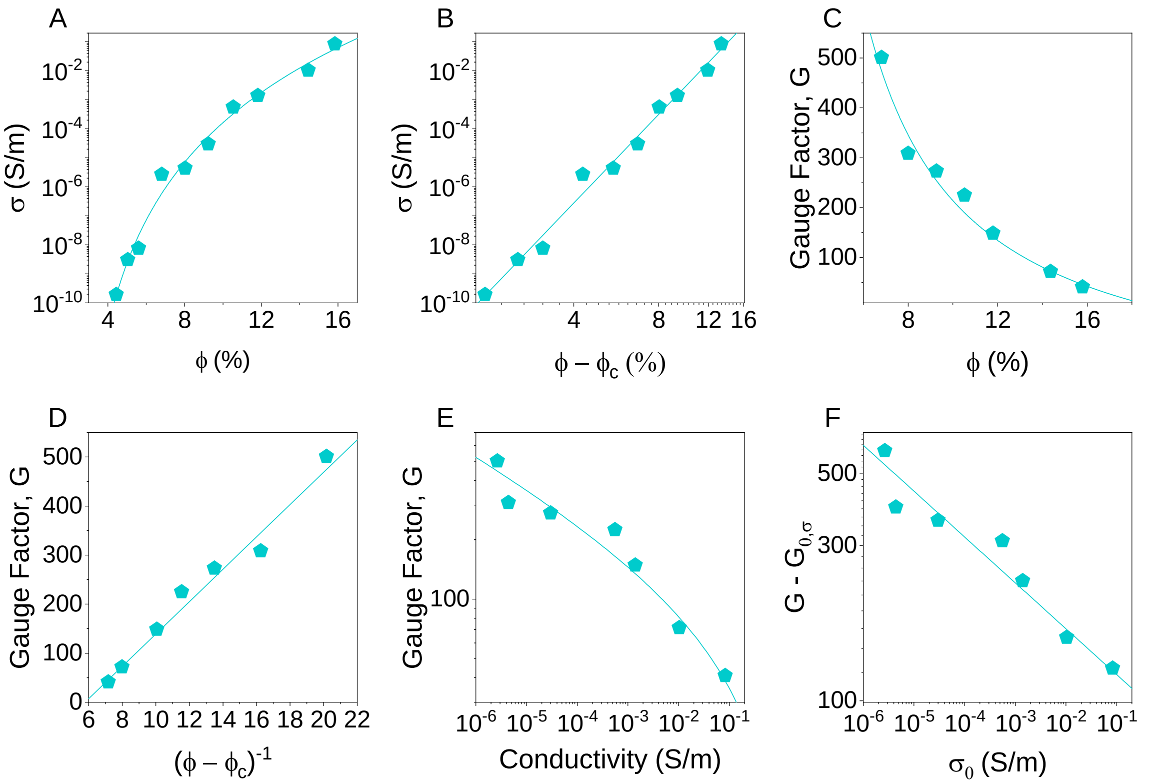

Figure S3: Fits to literature data, Boland et al.[4] The composite is comprised of a siloxane-based polymer matrix and graphene filler (G-putty). A-B) Zero-strain conductivity plotted as a function of A) Graphene volume fraction, ϕ, and B) zero-strain reduced volume fraction, $\phi-\phi_{c,0}$. The solid lines are fits to the percolation scaling law, Eq.1 (main text). $\sigma_{c,0} = 4.3 \times 10^7$ S/m, $\phi_{c,0} = 2.50\%$ and $t_0 = 10.2$ C-D) Gauge factor plotted versus C) graphene volume fraction, ϕ, and D) inverse of zero-strain reduced volume fraction, $(\phi-\phi_{c,0})^{-1}$. The solid lines are fits to equation 5a (main text). $\phi_{c,0} = 1.86\%$, $G_1 = 33$, $G_{0,\phi} = -190$ E-F) Gauge factor versus conductivity data plotted as G vs. σ_0 (E) and $G-G_{0,\sigma}$ vs. σ_0 (F). The solid line is a fit to Eq.5b (main text). $t = 7.1$, $\sigma_1 = 3.7\times10^{13}$ S/m, $G_{0,\sigma} = -85$

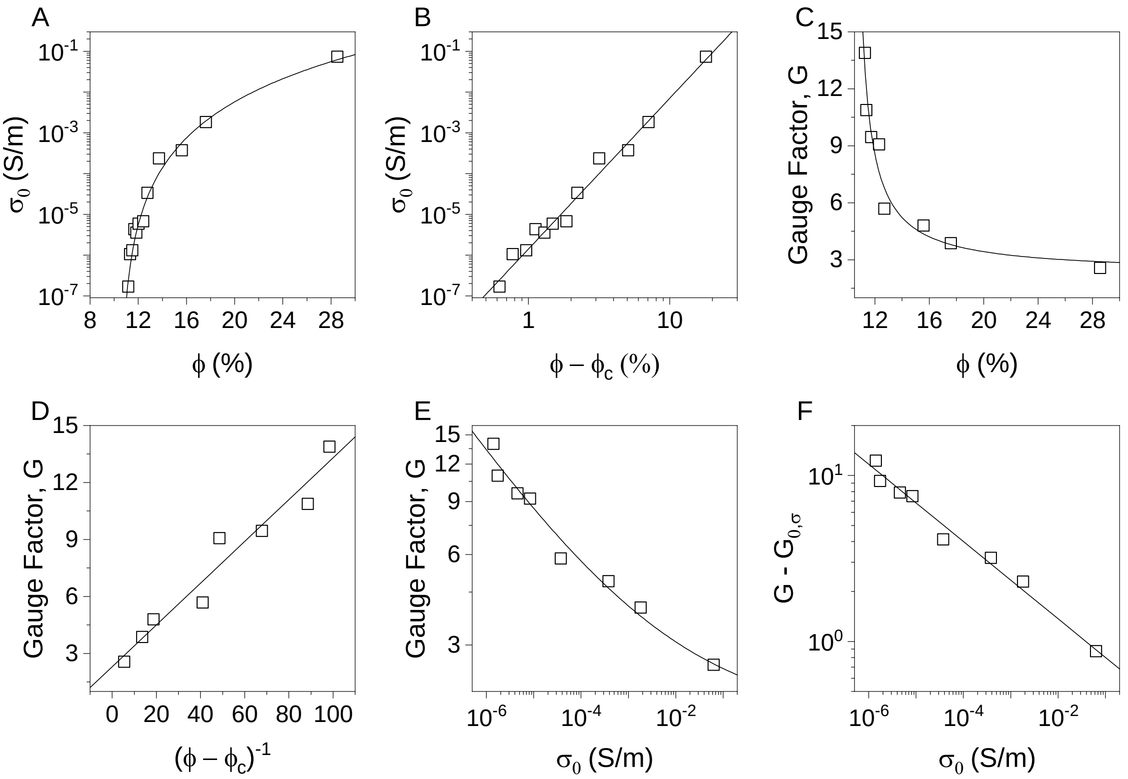

Figure S4: Fits to literature data, Carcia et al.[5] The composite is comprised of a borosilicate glass matrix and RuO_2 filler (Surface area 5 m^2/g). A-B) Zero-strain conductivity plotted as a function of A) RuO_2 volume fraction, ϕ, and B) zero-strain reduced volume fraction, ϕ-$\phi_{c,0}$. The solid lines are fits to the percolation scaling law, Eq.1 (main text). $\sigma_{c,0}$ = 34 S/m, $\phi_{c,0}$ = 10.55% and t_0 = 3.7 C-D) Gauge factor plotted versus C) graphene volume fraction, ϕ, and D) inverse of zero-strain reduced volume fraction, $(\phi-\phi_{c,0})^{-1}$. The solid lines are fits to equation 5a (main text). $\phi_{c,0}$ = 10.24% , G_1 = 0.11, $G_{0,\phi}$ = 2.3 E-F) Gauge factor versus conductivity data plotted as G vs. σ_0 (E) and G-$G_{0,\sigma}$ vs. σ_0 (F). The solid line is a fit to Eq.5b (main text). t_0 = 4.3, σ_1 = 0.039 S/m , $G_{0,\sigma}$ = 1.7

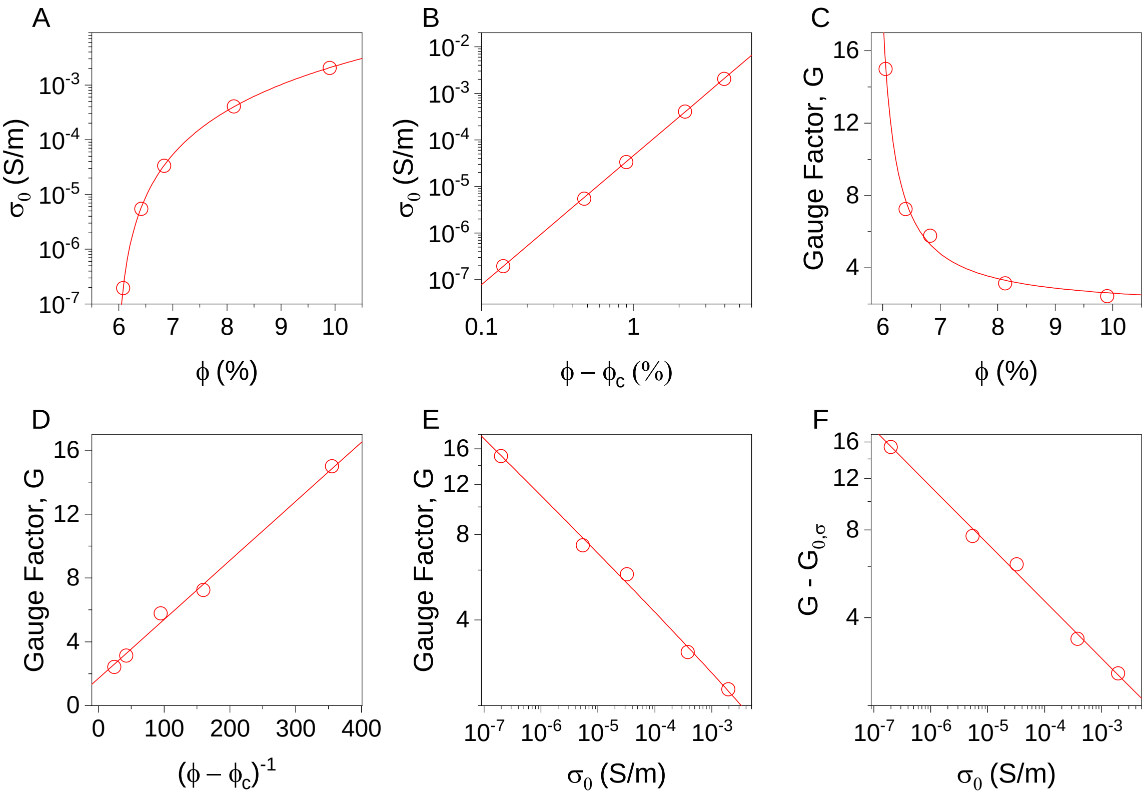

Figure S5: Fits to literature data, Carcia et al.[5] The composite is comprised of a borosilicate glass matrix and RuO_2 filler (Surface area 13 m^2/g). A-B) Zero-strain conductivity plotted as a function of A) RuO_2 volume fraction, ϕ, and B) zero-strain reduced volume fraction, ϕ-$\phi_{c,0}$. The solid lines are fits to the percolation scaling law, Eq.1 (main text). $\sigma_{c,0}$ = 16 S/m, $\phi_{c,0}$ = 5.94% and t_0 = 2.77 C-D) Gauge factor plotted versus C) graphene volume fraction, ϕ, and D) inverse of zero-strain reduced volume fraction, $(\phi-\phi_{c,0})^{-1}$. The solid lines are fits to equation 5a (main text). $\phi_{c,0}$ = 5.77% , G_1 = 0.038, $G_{0,\phi}$ = 1.7 E-F) Gauge factor versus conductivity data plotted as G vs. σ_0 (E) and G-$G_{0,\sigma}$ vs. σ_0 (F). The solid line is a fit to Eq.5b (main text). t_0 = 5.1, σ_1 = 0.23 S/m, $G_{0,\sigma}$ = -0.3

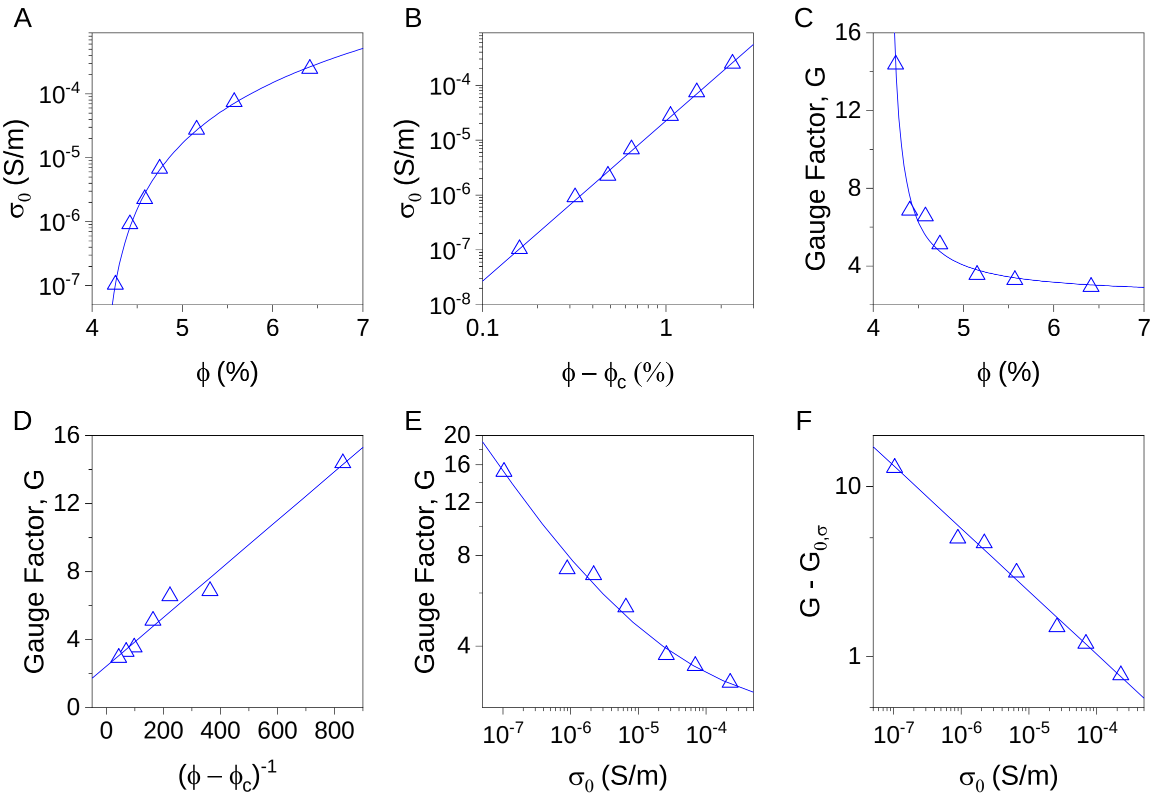

Figure S6: Fits to literature data, Carcia et al.[5] The composite is comprised of a borosilicate glass matrix and RuO_2 filler (Surface area 67 m^2/g). A-B) Zero-strain conductivity plotted as a function of A) RuO_2 volume fraction, ϕ, and B) zero-strain reduced volume fraction, ϕ-$\phi_{c,0}$. The solid lines are fits to the percolation scaling law, Eq.1 (main text). $\sigma_{c,0}$ = 16 S/m, $\phi_{c,0}$ = 4.10% and t_0 = 2.92 C-D) Gauge factor plotted versus C) graphene volume fraction, ϕ, and D) inverse of zero-strain reduced volume fraction, $(\phi-\phi_{c,0})^{-1}$. The solid lines are fits to equation 5a (main text). $\phi_{c,0}$ = 4.13% , G_1 = 0.0143, $G_{0,\phi}$ = 2.4 E-F) Gauge factor versus conductivity data plotted as G vs. σ_0 (E) and G-$G_{0,\sigma}$ vs. σ_0 (F). The solid line is a fit to Eq.5b (main text). t_0 = 2.71, σ_1 = 1.2×10^{-4} S/m, $G_{0,\sigma}$ = 2.25.

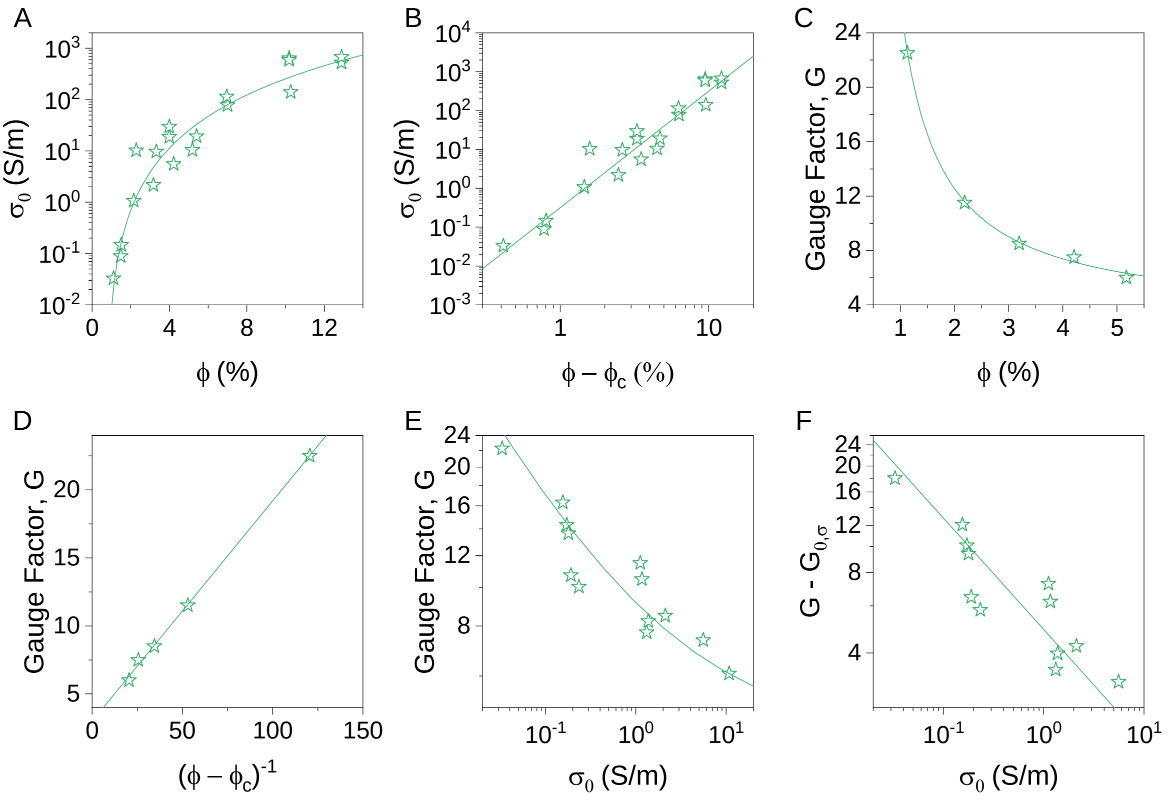

Figure S7: Fits to literature data, Hu et al.[21] The composite is comprised of an epoxy polymer matrix and carbon nanotube (CNT) filler. A-B) Zero-strain conductivity plotted as a function of A) CNT volume fraction, ϕ, and B) zero-strain reduced volume fraction, ϕ-$\phi_{c,0}$. The solid lines are fits to the percolation scaling law, Eq.1 (main text). $\sigma_{c,0} = 3.2 \times 10^5$ S/m, $\phi_{c,0} = 0.70\%$ and $t_0 = 3$ C-D) Gauge factor plotted versus C) graphene volume fraction, ϕ, and D) inverse of zero-strain reduced volume fraction, $(\phi-\phi_{c,0})^{-1}$. The solid lines are fits to equation 5a (main text). $\phi_{c,0} = 0.31\%$, $G_1 = 0.162$, $G_{0,\phi} = 3$ E-F) Gauge factor versus conductivity data plotted as G vs. σ_0 (E) and G-$G_{0,\sigma}$ vs. σ_0 (F). The solid line is a fit to Eq.5b (main text). $t_0 = 2.5$, $\sigma_1 = 53$ S/m, $G_{0,\sigma} = 4.2$.

Fit parameters

As described in the main text, standard strain sensor measurements lead to three distinct data sets (σ_0 vs. ϕ, G vs. ϕ and G vs. σ_0) that can be fit using equations 1, 5a and 5b yielding nine (main text) fit parameters as listed table 1. It is clear from table 1 that there is some degree of redundancy in these fit parameters. For example, the percolation threshold ($\phi_{c,0}$) can be extracted from fitting both σ_0 vs. ϕ and G vs. ϕ data while the percolation exponent (t_0) can be extracted from fitting σ_0 vs. ϕ and G vs. σ_0 data sets. In addition, inspection of equations 4b and 4c, in combination with equation 1, shows that the parameters $G_{0,\phi}$ and $G_{0,\sigma}$ are actually identical. Thus, under normal circumstances, it would not be necessary to fit all three data sets, with most researchers probably opting to fit only σ_0 vs. ϕ and G vs. ϕ. However, comparing fit parameters from all three data sets allows us to test the outputs of the model described above. To expand our data set, we examine not only the fit parameters obtained from figure 3 (main text) but also parameters found by fitting literature data (Fig S3-7).

We first plot the percolation threshold found by fitting the G vs. ϕ data using equation 5a with that obtained by fitting the σ_0 vs. ϕ data using equation 1 (Fig S8 A). Here we find extremely good agreement with all data very close to the line defining y=x. We then plot the percolation exponent found by fitting the G vs. σ_0 data using equation 5b with that obtained by fitting the σ_0 vs. ϕ data using equation 1 (Fig S8 B). Here we find the data points lying in the vicinity of the y=x line, although note some non-trivial deviations. For example, one of the graphene-polymer composites G-putty,[4] shows exponents of 10 and 7 which is a non-trivial deviation. However, by and large, we believe the agreement shown in figure S8 B is reasonable.

Shown in figure 3C are data for $G_{0,\phi}$ obtained by fitting G vs. ϕ data using equation 5a plotted versus $G_{0,\sigma}$ obtained by fitting G vs. σ_0 data using equation 5b. While these parameters should be equal as described above, we expect this data to show the greatest scope for deviation. The reason for this is that accurate values of these parameters require good data for G at high values of ϕ (and so σ_0). However, most papers do not report such data while aggregation effects can cause large errors. Yet, we do find reasonable agreement between these parameters with only two samples, both graphene-based giving significant deviation. Taken together, the results presented in figure S8 A-C give us confidence that the model above can accurately describe data. In addition, we note that equation 5 a-b show that large, positive values of $G_{0,\phi}$ and $G_{0,\sigma}$ lead to higher gauge factors. Interestingly, while most of the data is within 5 units of the origin,

two data points, both representing soft, graphene-siloxane composites lie far from the origin. One of these data points has large, positive values of $G_{0,\phi}$ and $G_{0,\sigma}$, which will act to boost G, the other has large negative values, which will act to decrease G.

Assuming the model is indeed accurate, it is worth asking what values are obtained for the parameters G_1 and σ_1. These parameters are plotted in figure S8 D and tend to scale with each other as would be expected for related (but unequal) parameters (see equations 4 b-c). Both parameters span a broad range with G_1 varying from ~0.01 to ~30 and σ_1 varying from 10^{-4} to 10^{13} S/m. Referring to equation 5 a-b, it is clear that large values of G_1 and σ_1 are required to achieve large gauge factors. With this in mind, it is interesting to note that RuO2 composites have the lowest values of G_1 and σ_1 while graphene-based composites have the highest with the CNT composite in the middle.

Taken together, the data in figure S8 C-D implies very large variations in sensing performance among different composites with considerable variations even in a single composite type (graphene-siloxane). This latter point suggests additional factor such as morphology may be important.

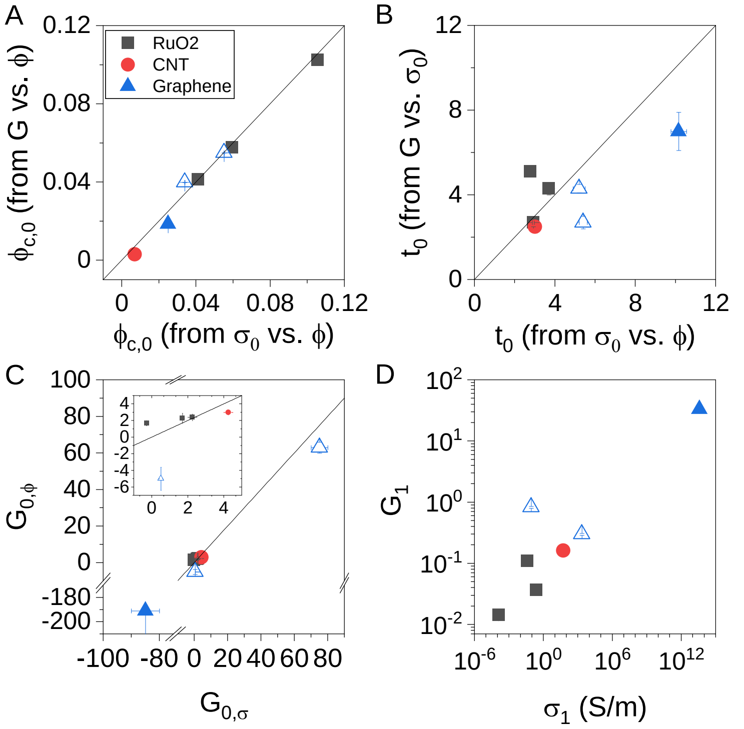

Figure S8: Parameters obtained by fitting data obtained in this work (open symbols) and literature data (solid symbols) to equations 1, 5a and 5b. A) Percolation threshold data obtained

by fitting data using equation 5a (G vs ϕ) and equation 1 (σ_0 vs ϕ) plotted against each other. B) Percolation exponent data obtained by fitting data using equation 5b (G vs σ_0) and equation 1 (σ_0 vs ϕ) plotted against each other. C) Fit parameters $G_{0\phi}$ and $G_{0\sigma}$, obtained by fitting data using equation 5a (G vs ϕ) and equation 5b (G vs σ_0) plotted against each other. D) Fit parameters G_1 and σ_1, obtained by fitting data using equation 5a (G vs ϕ) and equation 5b (G vs σ_0) plotted against each other.

Table S1: Various parameters obtained from linearized fits of data to Equations 1 (σ_0 vs. ϕ), 5a (G vs. ϕ) and 5b (G vs. σ_0).

Composite	Sylgard	G-putty	Boland et al[4]	Carcia et al[5] (5m^2/g)	Carcia et al[5] (13m^2/g)	Carcia et al[5] (67m^2/g)	Hu et al[21]
From fitting σ_0 vs. ϕ							
$\sigma_{c,0}$ (S/m)	4.6×10^7	3.3×10^3	4.3×10^7	34	16	16	3.2×10^5
$\phi_{c,0}$	0.055	0.034	0.025	0.1055	0.0594	0.041	0.007
t_0	5.1	5.4	10.2	3.7	2.77	2.92	3
From fitting G vs. ϕ							
$G_{0,\phi}$	-5	63	-190	2.3	1.7	2.4	3
G_1	0.32	0.83	33	0.11	0.038	0.0143	0.162
$\phi_{c,0}$	0.055	0.0399	0.0186	0.1024	0.0577	0.0413	0.0031
From fitting G vs. σ_0							
$G_{0,\sigma}$	0.5	75	-85	1.7	-0.3	2.25	4.2
σ_1 (S/m)	2200	0.084	3.7×10^{13}	0.039	0.23	1.2×10^{-4}	53
t_0	4.27	2.7	7.1	4.3	5.1	2.71	2.5

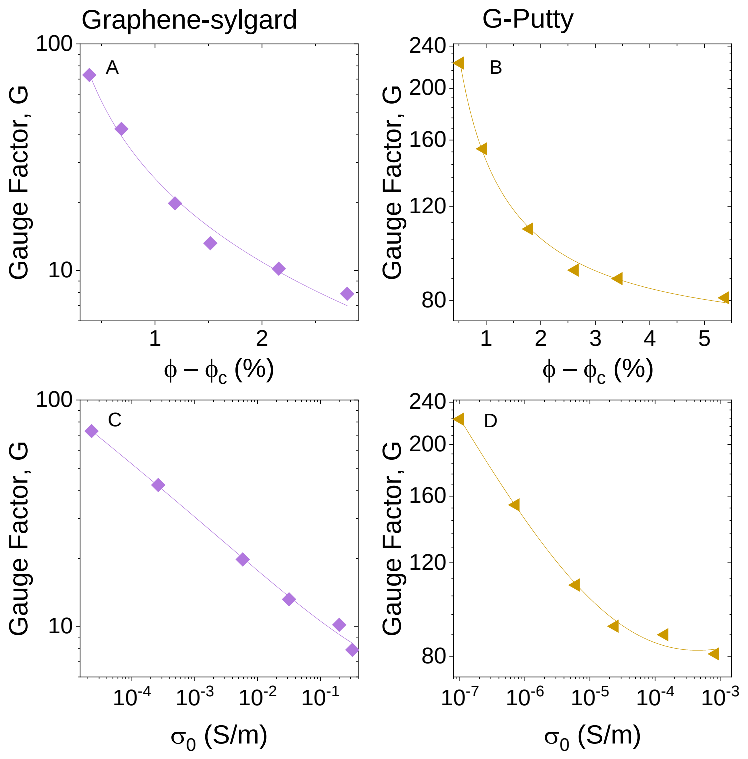

Figure S9: Values of G for graphene-sylgard and G-putty, plotted as a function of ϕ and σ_0. The data is fitted to Equations 4b,c here we have fixed the values of $\sigma_{c,0}$, $\phi_{c,0}$ and t_0 as per Table S1. Comparing these values (Table S2) to the expected values (determined obtained from linear fits at low strain to the percolation data presented in Figure 4 (C-H) main text) shows reasonable agreement in some values however others are far from the expected values from or have large errors. We mainly attribute this to a limited number of data points and suggest that producing composites with a larger number of filler volume fractions will yield more reliable derivative values.

Table S2: Values of $d \ln \sigma_c / d\varepsilon$, $d\phi_c / d\varepsilon$ and $dt / d\varepsilon$ for graphene-sylgard and G-putty composites determined by various fitting methods described in Figure S9.

Composite	Graphene-sylgard	G-putty
Expected Values		
$d \ln \sigma_c / d\varepsilon$	-4.4	-120
$d\phi_c / d\varepsilon$	0.07	0.5
$dt / d\varepsilon$	-4.2	-33
From fitting G vs. ϕ		
$d \ln \sigma_c / d\varepsilon$	0 ± 19	-66 ± 16
$d\phi_c / d\varepsilon$	0.06 ± 0.01	0.16 ± 0.01
$dt / d\varepsilon$	2 ± 5	-2 ± 5
From fitting G vs. σ_0		
$d \ln \sigma_c / d\varepsilon$	-16 ± 7	-205 ± 25
$d\phi_c / d\varepsilon$	0.068 ± 0.003	0.64 ± 0.04
$dt / d\varepsilon$	-6.3 ± 1.9	65 ± 9

References

1. Balberg, I.; Azulay, D.; Toker, D.; Millo, O., Percolation and tunneling in composite materials. *Int J Mod Phys B* **2004,** *18* (15), 2091-2121.
2. Johner, N.; Grimaldi, C.; Balberg, I.; Ryser, P., Transport exponent in a three-dimensional continuum tunneling-percolation model. *Phys Rev B* **2008,** *77* (17), 174204.
3. Arif, M. F.; Kumar, S.; Gupta, T. K.; Varadarajan, K. M., Strong linear-piezoresistive-response of carbon nanostructures reinforced hyperelastic polymer nanocomposites. *Composites Part A: Applied Science and Manufacturing* **2018,** *113*, 141-149.
4. Boland, C. S.; Khan, U.; Ryan, G.; Barwich, S.; Charifou, R.; Harvey, A.; Backes, C.; Li, Z.; Ferreira, M. S.; Möbius, M. E.; Young, R. J.; Coleman, J. N., Sensitive electromechanical sensors using viscoelastic graphene-polymer nanocomposites. *Science* **2016,** *354* (6317), 1257-1260.
5. Carcia, P. F.; Suna, A.; Childers, W. D., Electrical-Conduction and Strain Sensitivity in Ruo2 Thick-Film Resistors. *Journal of Applied Physics* **1983,** *54* (10), 6002-6008.
6. Chun, S.; Choi, Y.; Park, W., All-graphene strain sensor on soft substrate. *Carbon* **2017,** *116*, 753-759.
7. Christ, J. F.; Aliheidari, N.; Ameli, A.; Pötschke, P., 3D printed highly elastic strain sensors of multiwalled carbon nanotube/thermoplastic polyurethane nanocomposites. *Materials and Design* **2017,** *131*, 394-401.
8. Gao, J.; Wang, X.; Zhai, W.; Liu, H.; Zheng, G.; Dai, K.; Mi, L.; Liu, C.; Shen, C., Ultrastretchable Multilayered Fiber with a Hollow-Monolith Structure for High-Performance Strain Sensor. *ACS Applied Materials and Interfaces* **2018,** *10* (40), 34592-34603.
9. Kang, I.; Schulz, M. J.; Kim, J. H.; Shanov, V.; Shi, D., A carbon nanotube strain sensor for structural health monitoring. *Smart Materials and Structures* **2006,** *15* (3), 737.
10. Kim, J. H.; Hwang, J. Y.; Hwang, H. R.; Kim, H. S.; Lee, J. H.; Seo, J. W.; Shin, U. S.; Lee, S. H., Simple and cost-effective method of highly conductive and elastic carbon nanotube/polydimethylsiloxane composite for wearable electronics. *Scientific Reports* **2018,** *8* (1), 1-11.
11. Kim, J. Y.; Ji, S.; Jung, S.; Ryu, B. H.; Kim, H. S.; Lee, S. S.; Choi, Y.; Jeong, S., 3D printable composite dough for stretchable, ultrasensitive and body-patchable strain sensors. *Nanoscale* **2017,** *9* (31), 11035-11046.
12. Lee, W. S.; Kim, D.; Park, B.; Joh, H.; Woo, H. K.; Hong, Y.-K.; Kim, T.-i.; Ha, D.-H.; Oh, S. J., Multiaxial and Transparent Strain Sensors Based on Synergetically Reinforced and Orthogonally Cracked Hetero-Nanocrystal Solids. *Advanced Functional Materials* **2019,** *29* (4), 1806714-1806714.
13. Lee, Y. Y.; Kang, H. Y.; Gwon, S. H.; Choi, G. M.; Lim, S. M.; Sun, J. Y.; Joo, Y. C., A Strain-Insensitive Stretchable Electronic Conductor: PEDOT:PSS/Acrylamide Organogels. *Advanced Materials* **2016,** *28* (8), 1636-1643.
14. Liu, H.; Li, Y.; Dai, K.; Zheng, G.; Liu, C.; Shen, C.; Yan, X.; Guo, J.; Guo, Z., Electrically conductive thermoplastic elastomer nanocomposites at ultralow graphene loading levels for strain sensor applications. *Journal of Materials Chemistry C* **2015,** *4* (1), 157-166.
15. Paleo, A. J.; Hattum, F. W. J. v.; Pereira, J.; Rocha, J. G.; Silva, J.; Sencadas, V.; Lanceros-Méndez, S., The piezoresistive effect in polypropylene—carbon nanofibre composites obtained by shear extrusion. *Smart Materials and Structures* **2010,** *19* (6), 065013-065013.
16. Pham, G. T.; Park, Y. B.; Liang, Z.; Zhang, C.; Wang, B., Processing and modeling of conductive thermoplastic/carbon nanotube films for strain sensing. *Composites Part B: Engineering* **2008,** *39* (1), 209-216.
17. Wang, B.; Lee, B. K.; Kwak, M. J.; Lee, D. W., Graphene/polydimethylsiloxane nanocomposite strain sensor. *Review of Scientific Instruments* **2013,** *84* (10), 105005-105005.
18. Wang, S.; Zhang, X.; Wu, X.; Lu, C., Tailoring percolating conductive networks of natural rubber composites for flexible strain sensors via a cellulose nanocrystal templated assembly. *Soft Matter* **2016,** *12* (3), 845-852.
19. Wang, Y.; Yang, R.; Shi, Z.; Zhang, L.; Shi, D.; Wang, E.; Zhang, G., Super-elastic graphene ripples for flexible strain sensors. *ACS Nano* **2011,** *5* (5), 3645-3650.

20. Boland, C. S.; Khan, U.; Backes, C.; O'Neill, A.; McCauley, J.; Duane, S.; Shanker, R.; Liu, Y.; Jurewicz, I.; Dalton, A. B.; Coleman, J. N., Sensitive, high-strain, high-rate bodily motion sensors based on graphene-rubber composites. *ACS Nano* **2014,** *8* (9), 8819-8830.
21. Hu, N.; Karube, Y.; Arai, M.; Watanabe, T.; Yan, C.; Li, Y.; Liu, Y. L.; Fukunaga, H., Investigation on sensitivity of a polymer/carbon nanotube composite strain sensor. *Carbon* **2010,** *48* (3), 680-687.
